# From bench to bedside: Overcoming translational hurdles in nanoparticle research with pharmacokinetic modeling

**DOI:** 10.1002/btm2.70176

**Published:** 2026-09-08

**Authors:** Madison Parrot, Nuo Xu, Md Adnan, Joseph Cave, Hamidreza Ghandehari, Elizabeth Nance, Venkata Yellepeddi

**Affiliations:** ^1^ Division of Clinical Pharmacology, Department of Pediatrics Spencer Fox Eccles School of Medicine, University of Utah Salt Lake City Utah USA; ^2^ Department of Molecular Pharmaceutics Utah Center for Nanomedicine, College of Pharmacy, University of Utah Salt Lake City Utah USA; ^3^ Department of Chemical Engineering University of Washington Seattle WA USA; ^4^ Mathematics in Medicine Program, Houston Methodist Research Institute Houston Texas USA; ^5^ Physiology, Biophysics, and Systems Biology Program, Graduate School of Medical Sciences Weill Cornell Medicine New York New York USA; ^6^ Department of Biomedical Engineering, The John and Marcia Price College of Engineering University of Utah Salt Lake City Utah USA; ^7^ Department of Bioengineering University of Washington Seattle WA USA

**Keywords:** Nanomedicine, PBPK, pharmacokinetics, PK modeling, PopPK modeling, translation

## Abstract

Nanoparticles represent a major advancement in drug delivery, enhancing stability, solubility, and targeted delivery while reducing non‐specific toxicity. Despite promising preclinical results, few nanoparticle platforms have successfully translated to clinical use due to limited pharmacokinetic (PK) data, off‐target effects, manufacturing complexity, and a lack of long‐term studies. Emphasizing the pivotal role of PK in this development process is essential for harnessing the full therapeutic potential of nanoparticle applications in clinical practice. In this review, we introduce the concept of model‐informed nanoparticle development (MIND), an extension of model‐informed drug development (MIDD) principles to nanomedicine that integrates knowledge of nanoparticle interactions with biological systems and state‐of‐the‐art pharmacokinetic modeling to support evidence‐based decision‐making. Physiologically‐based PK (PBPK) models simulate the absorption, distribution, metabolism, and elimination (ADME) of nanoparticles within the body, incorporating physiological parameters to predict their PK behavior. Population‐based PK (PopPK) models utilize population variability to characterize nanoparticle PK across diverse patient groups, optimizing dosing strategies and personalized medicine approaches. Mechanistic models elucidate the intricate interactions between nanoparticles and biological systems, integrating cellular pathways and disease mechanisms to predict therapeutic outcomes and guide nanoparticle design. Through the MIND framework, these modeling approaches can enhance our understanding of nanoparticle PK, foster innovation in active agent delivery systems, and accelerate nanoparticle translation from research to clinical applications.

## Graphical abstract

Despite the promising applications of various nanoparticle compositions, shapes, sizes, and surface modifications, translation of these drug delivery systems has been limited. We propose leveraging pharmacokinetic modeling techniques to enable model‐informed nanoparticle development (MIND), supporting improved translation and facilitating informed clinical decision‐making.

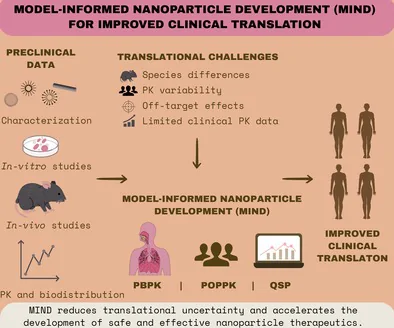

**Translational Impact Statement**

PK modeling can close the gap between preclinical and clinical studies for nanotherapeutics by providing physiologically‐based predictions for safety and exposure, dosing recommendations, and biodistribution patterns. PK modeling plays a critical role in improving nanotherapeutic translation and enabling safer, more effective treatments for patients.

## 1 INTRODUCTION

Nanoparticles are used in the delivery of therapeutic and diagnostic agents due to their ability to enhance stability, solubility, and targeted delivery while minimizing non‐specific toxicity. However, the same physicochemical features that confer therapeutic advantages also lead to complex, nonlinear, and system‐dependent PK behavior that is difficult to predict using empirical approaches. Nanocarrier compositions can include, but are not limited to, lipid‐based, inorganic, polymeric, or hybrid structures. The type of carrier used to incorporate an active agent can alter circulation time, cellular uptake, biodistribution, and clearance, all of which are key pharmacokinetic (PK) parameters that influence drug exposure and efficacy.^1^, ^2^, ^3^

The landscape of nanoparticle research is marked by a number of studies demonstrating the therapeutic efficacy of nanoparticles in preclinical models and the clinic.^4^, ^5^, ^6^ The coronavirus disease (COVID‐19) pandemic and implementation of nanoparticle‐based messenger ribonucleic acid (mRNA) COVID‐19 vaccines renewed interest and investment in nanoparticle therapies. The Pfizer/BioNTech and Moderna vaccines are protonated at acidic pH to allow for the complexation of lipid nanoparticles (LNPs) containing cationic lipids that protonate at acidic pH, allowing them to complex with mRNA.^7^ The cationic lipids also allow the LNPs to protect the negatively charged mRNA molecules from degradation and facilitate their entry into the cells.^8^ The weakly acidic cationic lipids are primarily protonated at endosomal pH (4.5‐6.5) to facilitate mRNA release. Biodistribution studies of these LNPs revealed predominant liver accumulation, a key determinant of both their efficacy and safety, highlighting the need for PK analyses to optimize delivery and minimize off‐target effects. It has been estimated that the first dose of the COVID‐19 vaccines alone prevented 14.4 million deaths in the first year of the pandemic.^9^ These findings emphasize the importance of quantitative PK analyses for understanding exposure‐response relationships and balancing efficacy with safety, particularly for nanoparticle systems with organ‐specific accumulation.

Despite the tremendous potential and the abundance of preclinical reports, including the success of the LNP‐based COVID‐19 vaccine, less than 100 nanoparticles have successfully navigated the complex journey from preclinical research to clinical application.^10^ Out of these nanoparticles, 2 LNPs, 0 polymeric nanoparticles, and 0 other forms of nanoparticles were approved after the first LNP‐based COVID‐19 vaccine (Comirnaty™) was approved.^11^ This discrepancy between the extensive preclinical exploration and the limited translation to clinical settings underscores the challenges and complexities inherent in advancing nanoparticle‐based technologies. A major contributor to this disconnect is the absence of integrative, predictive frameworks that systematically link nanoparticle design, biological interactions, and clinical exposure. While nanoparticles are used for both imaging and as diagnostics, and the combinatorial theranostic approach, our focus in this review lies on the therapeutic use of nanoparticles. The gap between preclinical potential and clinical reality necessitates a deeper understanding of the challenges involved in clinical translation, robust clinical trial design, and strategic collaborations between formulation, preclinical, and clinical disciplines to bridge this divide and unlock the full potential of nanoparticles as active agent carriers.

Important challenges in nanoparticle translation are their potential adverse effects due to undesired biodistribution leading to accumulation and immunogenicity, manufacturing scalability and reproducibility, and lack of optimal PK data.^12^, ^13^ For instance, the tendency of nanoparticles to accumulate in the liver and spleen due to uptake by the mononuclear phagocyte system (MPS) is a major limitation in achieving targeted delivery, necessitating PK studies to refine nanoparticle formulations and dosing regimens. To address these challenges, drug delivery scientists aim to enhance the safety, scalability, stability, batch‐to‐batch consistency, and predictability of nanoparticle formulations.

Integrating PK modeling into the preclinical‐to‐clinical pipeline remains underexplored and underutilized, representing lost potential in the field of nanoparticle research. PK modeling incorporates biodistribution data to predict nanoparticle behavior over time. This modeling methodology can address challenges in nanoparticle translation by predicting nanoparticle behavior and optimizing dosing regimens, thereby facilitating the transition from preclinical to clinical studies and supporting personalized treatment approaches by accounting for inter‐individual variability. PK modeling improves translation by predicting outcomes, optimizing experimental design, and reducing costs. Moreover, PK modeling can help address the translational gap for nanoparticles. For example, physiologically‐based pharmacokinetic (PBPK) modeling can integrate nanoparticle biodistribution data to predict human PK from preclinical species, refining first‐in‐human dose selection,^14^, ^15^, ^16^ a primary benefit of PK modeling. For nanoparticles, PBPK models are especially valuable for first‐in‐human predictions because they can incorporate size, charge, and MPS‐specific behavior, offering a more realistic and individualized prediction of how long particles will circulate, where they will accumulate, and how they are cleared.

PK modeling is widely accepted in the pharmaceutical industry to expedite the clinical translation of active agents and protect animal life.^17^, ^18^, ^19^, ^20^ Due to the complex structure of nanoparticles and the lack of standardized modeling methods, unlike those available for drugs such as small molecules or monoclonal antibodies, PK modeling is not as widely applied in the translation of nanoparticles. Most preclinical studies on new nanoparticle drug delivery systems prioritize evaluating efficacy and toxicity over understanding their PK and disposition. However, without a detailed understanding of nanoparticle biodistribution, PK models lack essential input parameters, such as tissue partition coefficients, organ‐specific distribution, and elimination parameters, limiting their predictive accuracy. This limited focus on mechanistic insights regarding nanoparticle PK and disposition poses challenges for effective modeling in the translational pathway. Despite these advantages, PK modeling for nanoparticles is often applied retrospectively or in isolation, limiting its impact on formulation design and translational decision‐making. A structured framework is therefore needed to formally integrate prior biological knowledge, experimental data, and mechanistic modeling throughout nanoparticle development.

To address these limitations, this review introduces the concept of model‐informed nanoparticle development (MIND). This structured framework integrates a priori knowledge of nanoparticle physicochemical properties, in vitro and in vivo biological interactions, and PK modeling to support evidence‐based decision‐making. Through the MIND framework, modeling becomes a prospective tool for translational extrapolation, population simulations, and dose guidance. By linking formulation designs directly to biological performance and clinical exposure, MIND enhances our understanding of nanoparticle PK, supports innovation in active agent delivery systems, and accelerates translation from preclinical research to clinical application.

This review analyzes the state of therapeutic nanoparticles in preclinical and clinical trials, challenges in nanoparticle translation to the clinic, and the current successes and ongoing potential of PK modeling in clinical translation. By integrating biodistribution data into PK models, researchers can better anticipate nanoparticle behavior, enhance model reliability, and refine dose selection for improved translational use in humans.

## 2 THERAPEUTICS APPROVED BY THE FDA AND IN CLINICAL TRIALS THAT CONTAIN NANOPARTICLES

We first assess the landscape of therapeutic nanoparticles approved by the Food and Drug Administration (FDA) and European Medicines Agency (EMA), and therapeutic nanoparticles administered via systemic administration routes in clinical trials. Of 75 FDA‐ or EMA‐approved nanomedicines, 25 (33.3%) are lipid‐based (Table 1), and 24 (32.0%) are polymer‐based nanoparticles (Table 2). As of February 2024, search results with the keyword “nanoparticle” on ClinicalTrials.gov produced 164 clinical trials for systemically administered therapeutic nanoparticles (Table S1) out of 640 clinical trials containing nanoparticles. LNPs are the most common vehicles for the systemic use of therapeutic nanomedicine. This may be due to advantages such as low immunogenicity, the ability to encapsulate hydrophilic and hydrophobic active agents, and ease of manufacturing and scalability.^21^, ^22^, ^23^ Despite their wide use, LNPs have drawbacks such as short systemic circulation time, instability in vivo, and active agent release during storage.^8^, ^24^ Polymeric nanoparticles are the next most widely used nanoparticles and have the advantages of potential biodegradability, controlled drug release, and cargo protection in biological environments.^25^ Similarly, polymeric nanoparticles have drawbacks, including potentially limited encapsulation efficiency for hydrophilic cargos and polydispersity that increases batch‐to‐batch variability.^26^, ^27^, ^28^ Polymeric lipid hybrid nanoparticles are an emerging platform for active agent delivery that combine the properties of lipid‐ and polymer‐based nanoparticles by having a polymeric core and lipids tethered with poly(ethylene glycol) (PEG) shell.^29^ Notably, PEG is a common component of many nanoformulations: 18 PEGylated nanoparticles are among the 75 approved nano‐formulations, constituting 24.0% of nano‐formulations.

**TABLE 1:** Lipid‐based therapeutic nanoparticles approved by the FDA or EMA.

Trade name	Particle type	Active ingredients	Application/Indication	Approval year	Particle size
AmBisome®	Liposomes	Amphotericin B	Fungal infections	EMA (1990), FDA (1997)	Less than 100 nm^1^, ^2^
Doxil® (Caelyx®)	PEGylated nano‐liposomes	Doxorubicin hydrochloride	Ovarian cancer; multiple myeloma; HIV‐associated KS	FDA (1995), EMA (1996)	Less than 100 nm^3^
Abelcet®	Lipids	Amphotericin B	Fungal infections	FDA (1995)	1600–11,000 nm^2^, ^4^
DaunoXome®	Liposomes composed of a lipid bilayer of distearoylphosphatidylcholine and cholesterol (2:1 molar ratio)	Daunorubicin citrate	Advanced HIV‐associated KS	FDA, EMA (1996)	45 nm^2^
Amphotec	Liposomes	Amphotericin B	Fungal infections	FDA (1996)	130 nm^2^
Caelyx®	PEGylated liposomes	Doxorubicin hydrochloride	Metastatic breast cancer; advanced ovarian cancer; HIV‐associated KS; multiple myeloma	EMA (1996)	90 nm^5^
Inflexal® V	Lecithin‐phospholipid liposomes	Haemagglutinin surface molecules of the influenza viruses	Influenza	EMA (1997)	About 150 nm^2^
Curosurf®	Liposomes	Poractant alfa	Respiratory distress syndrome in premature infants	FDA (1999)	Size distribution centered on 80 and 800 nm^6^
Visudyne®	Liposomes	Verteporfin	Age‐related macular degeneration	FDA, EMA (2000)	150–300 nm^2^
Myocet®	Liposomes	Doxorubicin hydrochloride	Metastatic breast cancer	EMA (2000)	180 nm^2^, ^7^
DepoCyt®	Liposomes	Cytarabine	Lymphomatous meningitis	EMA (2002), FDA (2007)	10–20 μm^2^
DepoDur®	Liposomes	Morphine sulfate	Post‐operative pain	FDA (2004), EMA (2006)	17–23 μm^2^
Mepact®	Liposomes	Mifamurtide	Osteosarcoma	EMA (2009)	2–3.5 μm^2^, ^8^
Exparel®	Liposomes	Bupivacaine	Pain management	FDA (2011)	24–31 μm^2^
Marqibo®	Sphingomyelin/Cholesterol liposomes	Vincristine sulfate	Acute lymphoblastic leukemia	FDA (2012). Approval is withdrawn as of May 2, 2022.	About 100 nm^2^, ^9^
Lipodox® (generic version of Doxil®/Caelyx®)	PEGylated nano‐liposomes	Doxorubicin hydrochloride	Ovarian cancer; metastatic breast cancer; HIV‐associated KS	FDA (2013)	180 nm^10^
Onivyde®	Liposomes	Irinotecan	Metastatic pancreatic cancer	FDA (2015)	110 nm^2^
Lipusu®	Liposomes	Paclitaxel	Breast cancer; lung squamous cell carcinoma	FDA (2016)	About 400 nm^11^
Vyxeos®	Liposomes	Daunorubicin and cytarabine	Acute myeloid leukemia or acute myeloid leukemia with myelodysplasia‐related changes	FDA (2017), EMA (2018)	About 100 nm^12^
Shingrix®	3‐O‐desacyl‐4′‐monophosphoryl lipid	Recombinant varicella zoster virus surface glycoprotein E antigen	Prevention of herpes zoster	FDA (2017), EMA (2018)	163 nm^13^
Onpattro® (patisiran)	PEGylated lipid	siRNA targeting transthyretin	Transthyretin‐mediated amyloidosis	FDA, EMA (2018)	60–100 nm
Arikayce®	Liposomes	Amikacin	Mycobacterium avium complex lung disease	FDA (2018)	About 300 nm
Comirnaty™ (Pfizer‐BioNTech Vaccine)	Lipids	Nucleoside‐modified mRNA	Covid‐19	FDA, EMA (2021)	60–65 nm^14^
Spikevax™ (Moderna COVID‐19 Vaccine)	Lipids	Nucleoside‐modified mRNA	Covid‐19	FDA, EMA (2022)	30–1000 nm^14^
Zolsketil®	PEGylated liposomes	Doxorubicin hydrochloride	Breast cancer; advanced ovarian cancer; multiple myeloma; HIV‐associated KS	EMA (2022)	About 100 nm^15^

Abbreviations: EMA, European Medicines Agency; FDA, Food and Drug Administration; HIV, Human Immunodeficiency Virus; KS, Kaposi's Sarcoma; mRNA, messenger RNA; N/A, no data available; siRNA, small interfering RNA.

**TABLE 2:** Polymer‐based therapeutic nanoparticles approved by FDA or EMA.

Trade name	Particle type	Active ingredients	Application/Indication	Approval year	Particle size
Diprivan®	Micellar nanoparticle	Propofol	Anesthesia or sedation	FDA (1989), EMA (2001)	30 nm^16^
Adagen®	Polymeric conjugate (PEGylated)	Adenosine deaminase	Severe combined immunodeficiency disease	FDA (1990)	N/A
Oncaspar®	Polymeric conjugate (PEGylated)	Asparaginase	Acute leukemia; hypersensitivity to asparaginase	FDA (1994), EMA (2016)	50–200 nm
Apelea®	Micellar paclitaxel	Paclitaxel	Ovarian cancer	2018 (EMA)	20–30 nm
Copaxone®	Polypeptides	Glatiramer acetate	Multiple sclerosis	FDA (1996), EMA (2016)	Around 5 and 111 nm^17^
Renagel®	Sevelamer (consists of polyallylamine that is crosslinked with epichlorohydrin)	Sevelamer hydrochloride	Hyperphosphatemia caused by chronic kidney disease	FDA, EMA (2000)	23 μm^18^
PegIntron®	Polymeric conjugate (PEGylated)	Interferon alfa‐2a	Chronic hepatitis C	EMA (2000), FDA (2001)	N/A
Pegasys®	Polymeric conjugate (PEGylated)	Interferon alfa‐2a	Chronic hepatitis C; chronic hepatitis B	FDA, EMA (2002)	0.5–23 nm^19^
Zevalin®	Polymeric conjugate	*β*‐emitting isotope, (90)Y, linked to the anti‐CD20 monoclonal antibody (mAb), rituximab	Non‐Hodgkin's lymphoma	FDA (2002) (Discontinued), EMA (2004)	N/A
Somavert®	Polymeric conjugate (PEGylated)	Growth hormone receptor antagonist	Acromegaly	EMA (2002), FDA (2003)	N/A
Avinza®	Polymer beads constituted by ammonium‐methacrylate copolymers	Morphine sulfate	Moderate to severe pain	FDA (2002) (Discontinued)	N/A
Neulasta®	Polymeric conjugate (PEGylated)	Filgrastim	Neutropenia caused by receiving chemotherapy	FDA (2002)	6 nm^20^
Estrasorb™	Micellar nanoparticle	Estradiol hemihydrate	Moderate to severe hot flashes after menopause	FDA (2003)	About 1 μm
Macugen®	Polymeric conjugate (PEGylated)	Anti‐vascular endothelial growth factor aptamer	Wet age‐related macular degeneration	FDA (2004)	N/A
Genexol‐PM®	Micellar nanoparticle	Paclitaxel	Lung cancer	FDA (2007)	24 nm^21^
Renvela®	Sevelamer (consists of polyallylamine that is crosslinked with epichlorohydrin)	Sevelamer carbonate	Hyperphosphatemia caused by chronic kidney disease	FDA (2007), EMA (2009)	25–65 μm
Mircera®	Polymeric conjugate (PEGylated)	Epoetin beta	Anemia	EMA (2007), FDA (2018)	N/A
Cimzia®	Polymeric conjugate (PEGylated)	Tumor necrosis factor (TNF) blocker	Crohn's disease; arthritis; active ankylosing spondylitis; plaque psoriasis	FDA (2008), EMA (2009)	9 nm
Krystexxa®	Polymeric conjugate (PEGylated)	Porcine‐like recombinant uricase	Gout	FDA (2010)	N/A
Plegridy®	Polymeric conjugate (PEGylated)	Interferon beta 1a	Multiple sclerosis	FDA (2014)	N/A
Adynovate®	Polymeric conjugate (PEGylated)	Recombinant antihemophilic factor	Bleeding episodes; Perioperative management	FDA (2015)	N/A
VivaGel® BV	Dendrimer based nanoparticulate	Astodrimer sodium	Bacterial vaginosis	FDA (2015)	9 nm
Rebinyn®	Polymeric conjugate (Glycopegylated)	Recombinant DNA‐derived coagulation factor IX	Hemophilia B	FDA (2017)	N/A
Zilretta®	PLGA (LA:GA = 75:25) microspheres	Triamcinolone acetonide	Osteoarthritis pain of the knee	FDA (2017)	About 45 μm^22^
Apealea®	Micellar nanoparticle	Paclitaxel	Platinum‐sensitive epithelial ovarian cancer; primary peritoneal cancer; fallopian tube cancer	EMA (2018)	20–60 nm

Abbreviations: DNA, deoxyribonucleic acid; EMA, European Medicines Agency; FDA, Food and Drug Administration; PLGA, poly(lactic‐co‐glycolic acid); N/A, no data available.

Blank polymeric nanoparticles can trigger an immune response and impact disease state, even without an active pharmaceutical agent.

Besides LNPs and polymer‐based nanoparticles, protein‐based nanoparticles (5.3%), nanocrystals (20.0%), and inorganic nanoparticles (9.3%) comprise the remaining third of therapeutics containing nanoparticles in the global market (Table 3). Inorganic nanoparticles are generally confined by the short systemic circulation time and in some cases poor biocompatibility. Some nanoparticles are more frequently used for imaging‐based applications,^29^, ^30^ and therefore are not a focus of this review.

**TABLE 3:** Other therapeutic nanoparticle‐based delivery systems approved by FDA or EMA.

Trade name	Particle type	Active ingredients	Application/Indication	Approval year	Particle size
Protein‐based nanoparticles					
Ontak®	Protein nanoparticle	Recombinant DNA‐derived cytotoxic protein composed of diphtheria toxin and human interleukin‐2 (IL‐2)	Cutaneous T‐cell lymphoma	FDA (1999)	N/A
Abraxane	Albumin‐bound particle	Paclitaxel	Advanced cancer of breast, lung, or pancreas	FDA (2005), EMA (2008)	130 nm^23^
Rapamune®	Protein‐bound particle	Sirolimus (rapamycin)	Kidney transplant; lymphangioleiomyomatosis	EMA (2001), FDA (2010)	250 nm
Fyarro™	Albumin‐bound particle	Sirolimus (rapamycin)	Perivascular epithelioid cell tumor	FDA (2021)	Less than 100 nm
Nanocrystals					
Ritalin LA®	Nanocrystalline	Methylphenidate	Attention deficit hyperactivity disorder	FDA (2002)	N/A
Zanaflex®	Nanocrystalline	Tizanidine hydrochloride	Spasticity	FDA (2002)	N/A
OsSatura®	Nanocrystalline	Hydroxyapatite	Bone voids or defects	FDA (2003)	N/A
Emend®	Nanocrystalline	Aprepitant	Nausea and vomiting caused by chemotherapy	FDA, EMA (2003)	120 nm
Vitoss®	Nanocrystalline	Bovine skin collagen (type I) with a porous tricalcium phosphate inorganic phase	Bony voids or gaps arise due to trauma, tumors or osteolysis	FDA (2003)	100 nm
Ostim®	Nanocrystalline	Hydroxyapatite	Bone defect	FDA (2004)	19 nm
TriCor®	Nanocrystalline	Fenofibrate	High levels of cholesterol and triglycerides	FDA (2004)	412 nm^8^
Focalin XR®	Nanocrystalline	Dexmethylphenidate hydrochloride	Attention deficit hyperactivity disorder	FDA (2005)	N/A
Megace ES®	Nanocrystalline	Megestrol acetate	Loss of appetite and wasting syndrome in people with AIDS	FDA (2005)	Less than 2000 nm
NanOss®	Nanocrystalline	Hydroxyapatite granules	Advanced bone graft substitute	FDA (2005)	15–100 nm
Invega®	Nanocrystalline	Paliperidone	Schizophrenia	FDA (2006)	N/A
Ivemend®	Nanocrystalline	Fosaprepitant dimeglumine	Nausea and vomiting caused by chemotherapy	FDA, EMA (2008)	N/A
Invega Sustenna®	Nanocrystalline	Paliperidone palmeitate	Schizophrenia	FDA (2009)	2.5 μm
EquivaBone®	Nanocrystalline	Calcium phosphate, carboxymethyl cellulose and human demineralized bone matrix	Bone voids or defects	FDA (2009)	Less than 100 nm
Ryanodex®	Nanocrystalline	Dantrolene sodium	Malignant hyperthermia	FDA (2014)	407 nm
Inorganic nanoparticles					
Infed® (CosmoFer®)	Iron dextran colloid		Iron deficiency anemia and iron deficiencies	FDA (1992)	About 15 nm^24^
Dexferrum®	Iron dextran		Iron deficiency anemia and iron deficiencies	FDA (1996)	31.5–36.5 nm^25^
Ferrlecit®	Sodium ferric gluconate		Iron deficiency anemia and iron deficiencies	FDA (1999), EMA (2013)	12 nm^26^
Venofer®	Iron sucrose nanocolloidal		Iron deficiency anemia in people with kidney disease	FDA (2000)	7 nm^25^
Feraheme®	Iron oxide nanoparticles (coated with polyglucose sorbitol carboxymethylether)		Iron deficiency anemia	FDA (2009)	17–31 nm^25^
Ferinject®	Ferric carboxymaltose nanoparticle (Ferric hydroxide core with a carbohydrate shell)		Iron deficiency anemia and iron deficiencies	FDA, EMA (2013)	23–25 nm^27^
Hensify®	Hafnium oxide nanoparticles		Locally advanced soft tissue sarcoma	EMA (2019)	50 nm^12^

Abbreviations: AIDS, Acquired Immune Deficiency Syndrome; EMA, European Medicines Agency; FDA, Food and Drug Administration; N/A, no data available.

Despite the demonstrated advantages of nanoparticles for therapeutic delivery, there remains a lack of preclinical to clinical translation. We analyze key barriers, including gaps in preclinical PK data, challenges in nanoparticle manufacturing and synthesis, limited analysis of off‐target and long‐term toxicity, and the untapped potential of PK modeling to bridge the translational divide.

## 3 CHALLENGES IN TRANSLATION OF NANOPARTICLE‐BASED DRUG DELIVERY SYSTEMS

**FIGURE 1:**
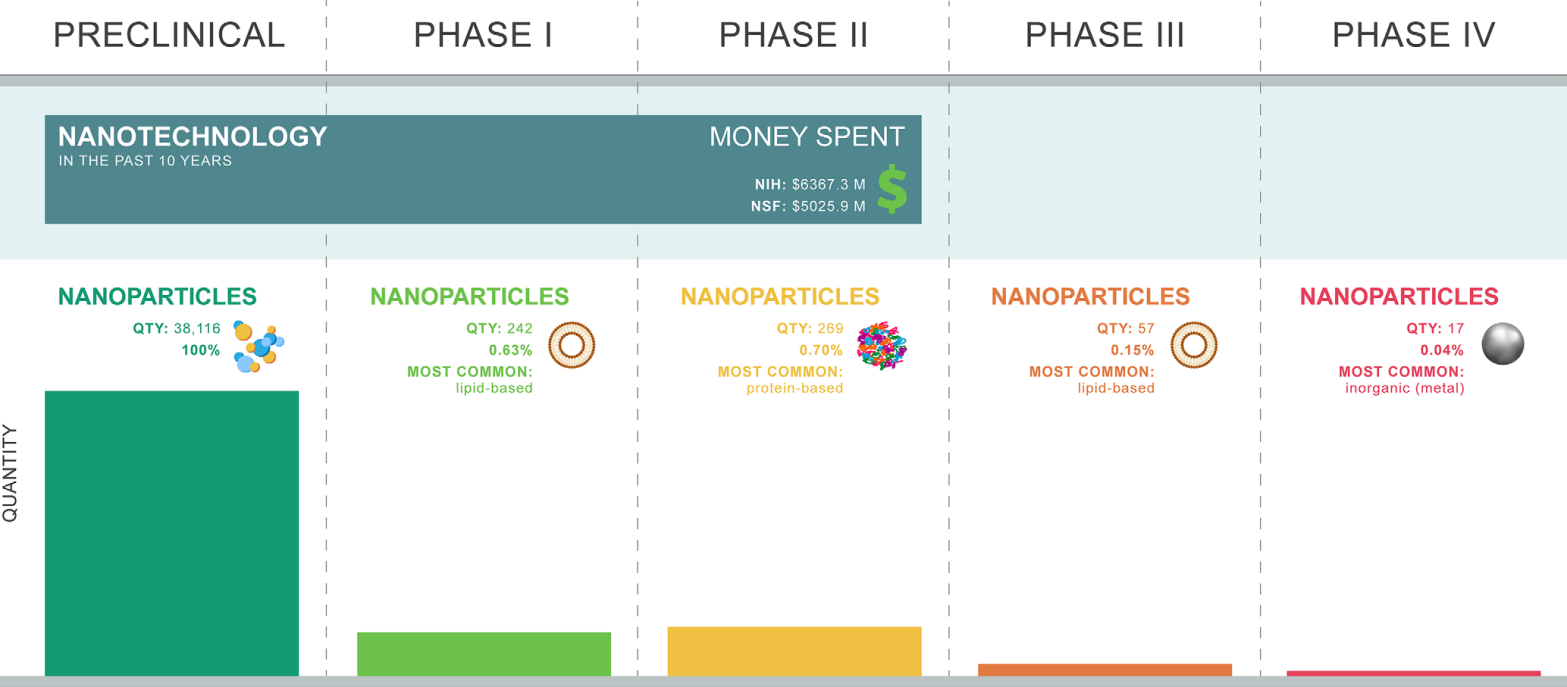
Current nanoparticle types and quantity in the various clinical trial stages. Note that bar heights are not drawn to scale and should not be interpreted quantitatively.

Only 20% of nanoparticle‐based delivery systems assessed in preclinical studies have been considered for translational investigation, and the actual clinical translation rate is even lower due to stability, scalability, efficacy, and toxicity issues.^31^ Most nanoparticle‐based drugs in clinical trials are in Phase I (242) or Phase II (269). Between 2001 and 2024, 57 nanoparticle‐based drugs successfully moved from Phase I and II to Phase III, and only 17 moved from the first three Phases to enter Phase IV (Figure 1). The majority of failures occur in Phase II trials due to the lack of efficacy or toxicity.^32^, ^33^, ^34^ In this section, we explore the need for robust PK data, the potential for and effect of off‐target effects, the necessity for high‐throughput long‐term toxicity studies, the impact of manufacturing and scale‐up on formulation parameters, and the pivotal role of PK in understanding and advancing nanoparticle‐drug applications.

**FIGURE 2:**
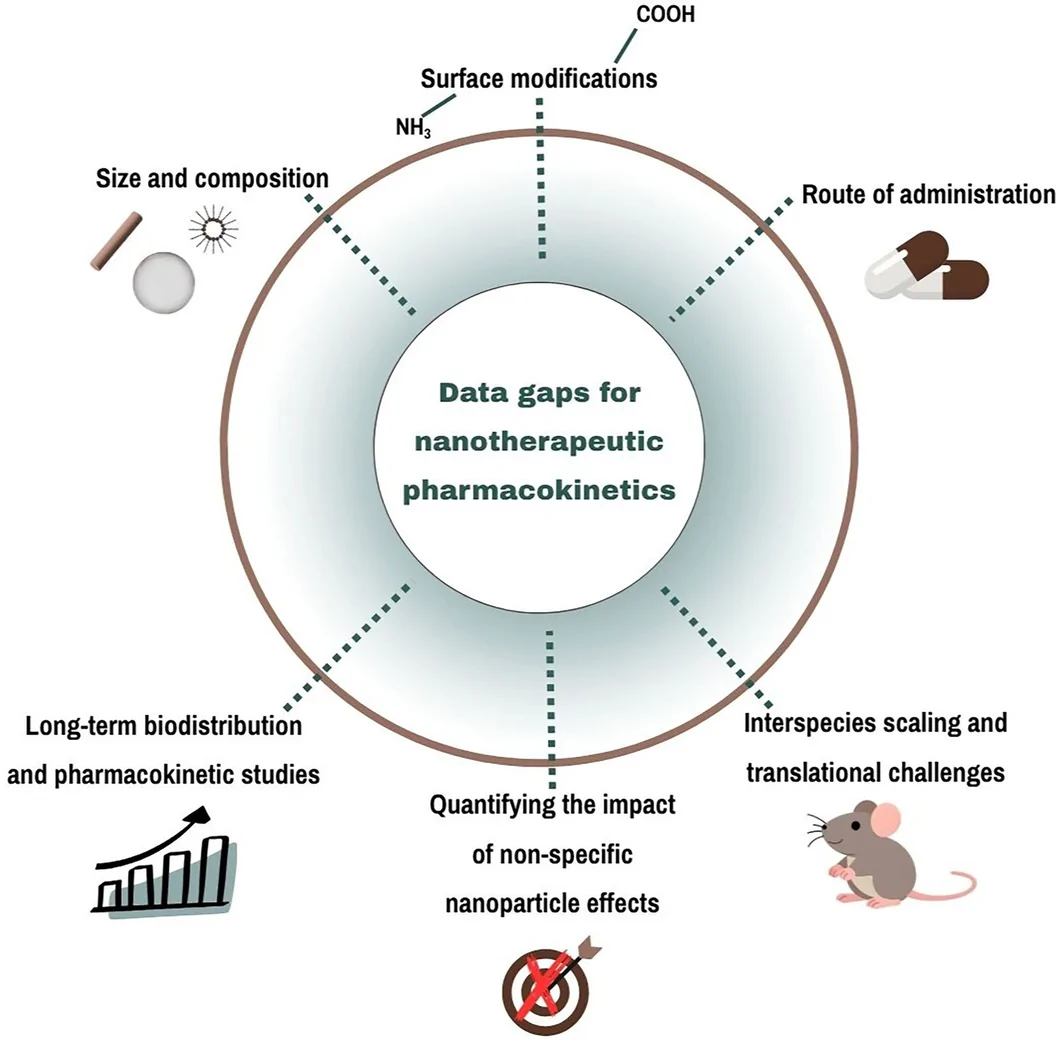
Overview of key data gaps in nanotherapeutic pharmacokinetics, including physicochemical properties, administration routes, biodistribution, and translational challenges.

Key data gaps necessary for successful nanoparticle translation are summarized in Figure 2.

### 3.1 Data gaps for nanotherapeutic PK

Lack of optimal PK data can hinder translating promising preclinical results to clinical efficacy. Integrating PK studies into the early stages of development can provide valuable insights for optimizing formulations and predicting clinical outcomes. Preclinical PK studies of nanoparticles are often performed with the active agent‐loaded nanoparticle rather than the nanoparticle itself, limiting the ability to decouple the effects of individual components.^35^ To make clinical predictions, it is essential to conduct preclinical PK studies on the nanoparticle alone, the active agent alone, and the combined formulation. Furthermore, a comprehensive dataset is crucial to accurately represent how nanoparticle physicochemical properties, such as size, size distribution, and surface modification, affect PK. The route of administration can influence the behavior and PK of nanoparticles, as can the host species in which the study is conducted.^36^, ^37^, ^38^ Accounting for these factors is critical to gain a holistic understanding of nanoparticle interactions within biological systems.

Kumar et al. compiled data from over 2000 datasets to elucidate patterns in nanoparticle PK in mice and determined that there is insufficient in vivo and in vitro data for proper PK characterization and modeling, including the fact that 65% of datasets had fewer than four time points.^39^ Long‐term toxicity studies are crucial for assessing the safety of nanoparticle‐based therapies over extended periods; however, challenges lie in conducting these studies efficiently and cost‐effectively.

Traditional in vivo studies of nanoparticles describe organ‐level biodistribution, rather than quantitatively evaluating PK parameters. These studies describe where the NPs go but lack the mathematical rigor to describe how fast they get there, how long they stay, and how they are cleared. PK studies go beyond a biodistribution snapshot at set time points to describe key quantitative parameters over time. Researchers still widely disagree on the interactions of nanoparticles with tissues, leading to inaccurate in vivo predictions, highlighting the need for PK studies to confirm predictions.^40^ Understanding nanoparticle clearance and half‐life allows for rational dose selection to avoid toxicity. PK data also enables inter‐species scaling for clinical translation and is required by regulatory agencies for approval. Factors such as biodistribution, clearance rates, and systemic exposure influence the therapeutic efficacy and safety of nanoparticle‐based active agents. It has been well established that physicochemical properties of nanoparticles, such as nanoparticle size, size distribution, geometry, and surface chemistry, including surface charge, impact their PK.^41^, ^42^, ^43^, ^44^, ^45^, ^46^

#### 3.1.1 Size and composition

While biodistribution describes the dispersion in the body and PK describes the time‐dependent absorption, distribution, metabolism, and elimination (ADME) of a drug or nanoparticle, one of the governing components of both is clearance. It was previously thought that nanoparticles over 8 nm in diameter cannot permeate the glomerular filtration barrier,^47^ but this has been disproven. Although dependent on composition and flexibility, generally nanoparticles that are 8–10 nm in diameter are easily removed from the body through the kidney's glomeruli.^48^, ^49^ Mesoporous silica and polymeric nanoparticles up to 200 nm have been detected in urine.^50^, ^51^ The same phenomenon applies to endothelial and epithelial transport. For example, nanoparticles up to 114 nm coated in PEG have been shown to cross the blood–brain barrier,^52^ despite the space between brain cells only being 20–60 nm.^53^ Similarly, smaller nanoparticles are considered to have better oral absorption despite 500 nm polymeric nanoparticles permeating the epithelium.^54^ To prevent being trapped by liver fenestrae and spleen sinusoids (size≈150–200 nm), the diameter of particles should be <100 nm, generally.^55^ Nanoparticles tend to accumulate in the liver and decrease clearance, although accumulation can depend on size, composition, and surface modifications. Nanoparticles >1 μm have increased clearance rates and decreased systemic half‐life because they are physically trapped by capillaries which drives rapid engulfment of these particles by phagocytic cells.^56^ Due to this variation, it is important to consult the literature for size‐ and composition‐specific filtration, permeability, and uptake for the nanoparticle in question.

Size‐based biodistribution also depends on nanoparticle composition; therefore, more PK studies are needed to elucidate these patterns.^57^, ^58^ PK could depend as much on composition as on size, so when new materials are developed, nanoparticles of the same composition but varying sizes should be created and tested for PK^59^ to better quantify the relationship between size, composition, and PK profiles. The shape of the nanoparticle will also affect the PK: spherical nanoparticles and worm‐like micelles have longer circulation times (and *t*_1/2_) compared to non‐spherical ones, but specific PK patterns related to shape and geometry have not yet been established; hence the need for more robust PK data.^58^, ^60^, ^61^

Pathological conditions can notably alter physiological processes like blood extravasation and, therefore, PK. For instance, the abnormal vasculature of tumors increases permeability, allowing drugs and small nanoparticles to extravasate and accumulate in tumors. This can reduce the maximum plasma concentration (*C*_max_) while increasing drug exposure in the tumor microenvironment, emphasizing the importance of PK studies in disease models like tumor‐xenograft models of cancer.^62^ Furthermore, extravasation in healthy tissues is size‐dependent, and achieving size‐specific uptake requires nanoparticle uniformity; however, due to the inherent polydispersity of many nanoparticles, particularly organic nanoparticles, comprehensive PK data across a range of sizes is essential to improve translational outcomes.^63^ There is an additional challenge of PK predictions in that nanoparticles are often assumed to be spherical and homogenously sized, which has resulted in the field defining an acceptable polydispersity index less than 0.2.^64^

#### 3.1.2 Surface modifications

Surface modification impacts PK by altering nanoparticle stability, interfacial interactions, systemic circulation time, and uptake by the reticuloendothelial system (RES), as well as reducing particle aggregation and cytotoxicity.^65^, ^66^, ^67^ The most common surface modification technique for injectable active‐agent delivery systems involves PEGylation. PEGylation extends circulation time by sterically protecting nanoparticles and increasing their hydrophilicity, which prevents aggregation and potentially reduces kidney clearance, although this is debated.^7^, ^68^, ^69^, ^70^, ^71^ Balancing the density and distance between grafted PEG molecules is crucial to reducing protein absorption that results in opsonization, while ensuring effective delivery of the nanoparticle's payload. Opsonization changes a nanoparticle's in vivo hydrodynamic diameter and surface presentation and increases the likelihood of non‐specific cellular interactions and macrophage recognition.^71^, ^72^ The increased macrophage uptake accelerates clearance, limiting drug accumulation at the target site and reducing efficacy. Additionally, interactions between surface‐bound nanoparticles and dysopsonins are a driver of the increased circulation time of PEGylated nanoparticles, and decreased ability to extravasate.^72^, ^73^ While the PK of PEGylated nanoparticles has been more extensively studied, comparative PK studies on nanoparticles with different surface modifications beyond PEG remain scarce. They are essential to quantify their impact on PK behavior.

Surface modifications can also alter surface charge. For many administration routes intended for systemic delivery, slightly negative to near‐neutral surface charges have the greatest benefit for active agent delivery.^52^, ^74^, ^75^ This is mostly attributed to positively charged nanoparticles maintaining strong interactions with plasma proteins and macrophages, leading to rapid clearance/short half‐life and increased toxicity due to their interactions with blood components. While positively charged nanoparticle applications are mainly focused on nucleic acid or bacterial cell delivery, cationic chitosan nanoparticles have been used in wound dressings to enhance interactions with the negatively charged bacterial membrane that the treatment is intended to kill.^76^ In some applications, a greater magnitude surface charge is more favorable. For example, vaccine nanoparticles leverage electrostatic interactions as they promote the adsorption of cargo. In addition to the previously discussed effect of surface modifications on reduced opsonization and clearance of nanoparticles, surface modification can alter the hydrophilicity/hydrophobicity thereby changing nanoparticle clearance via phagocytosis or the release kinetics of the cargo by pH‐ or enzyme‐sensitive surface agents. Therefore, understanding the relationship between surface modification and PK properties is essential for accurately predicting nanoparticle behavior, accounting for administration routes where different biological barriers may be present.

#### 3.1.3 Route of administration

The route of administration is a critical factor in nanoparticle translation that must be accounted for when developing PK datasets. Nanoparticles can be administered through transdermal, implantation, oral, inhalation, nasal, ocular, rectal, intragastric, sublingual, buccal, and intravenous (IV).^77^, ^78^, ^79^, ^80^, ^81^, ^82^, ^83^ IV administration leads to broad biodistribution with a short distribution phase and higher clearance by the RES. Oral administration introduces a bioavailability barrier due to enzymatic breakdown in the gastrointestinal tract, first‐pass metabolism in the liver, and epithelial transport barrier, but also allows pH‐dependent delivery or controlled release. After absorption from oral administration, nanoparticles enter systemic circulation, although degradation and limited absorption through the intestinal epithelium will lower bioavailability. For example, transdermal and inhalation administration can allow for local targeted delivery to the epidermis, nasal mucosa, and alveoli. Nanoparticles administered by inhalation can be rapidly distributed through the body if they cross the alveolar‐capillary membrane, which can be advantageous for drugs requiring fast onset of action. Achieving injectability, especially for systemic delivery, requires addressing issues such as particle size, stability during injection, and the prevention of aggregation. Due to injection‐site inflammation and increased immune response concerns, nanoparticles carrying cytotoxic payloads cannot be delivered intramuscularly or subcutaneously.^84^ The route of administration determines the biodistribution and clearance of nanoparticles but also influences their interaction with physiological barriers, immune responses, and delivery efficiency. Overcoming these challenges ensures effective and controlled delivery of nanoparticles to the target site. However, the route of administration may vary between the preclinical and clinical scenarios, depending on the species used in these studies.

In addition, the intended route of administration of nanoparticles in humans may differ from the route used in preclinical research. For example, while it is relatively straightforward to administer drugs directly into a brain tumor in preclinical models via intracranial injection, this method is often not feasible in clinical settings. Instead, more clinically practical routes, such as intrathecal or IV administration, could be used. In such scenarios, a wealth of preclinical data generated from intracranial injections may seem incompatible with clinical applications. However, PK modeling can bridge this gap by using preclinical intracranial injection data to build mechanistic models of nanoparticle disposition. These models can then be adapted to predict the PK for alternative routes of administration, facilitating the translation of preclinical findings to clinical applications. A practical example of this is the drug buprenorphine, used sublingually in neonates and intravenously in adults. When making a dose recommendation for a neonate, it is important to be very accurate, but PK data in this sensitive neonatal population are very sparse. Therefore, a PBPK model was developed from readily available adult IV data and converted to a neonatal PBPK model for dosing predictions.^16^

#### 3.1.4 Interspecies scaling and translational challenges

Interspecies differences including accessible administration routes and different physiological parameters that influence ADME, contribute to why many studies in animal models fail to translate to human models, especially in nanomedicine.^62^, ^85^, ^86^, ^87^, ^88^, ^89^, ^90^ Crommelin et al. in 2013 critiqued the use of animal models in nanoparticle research for lacking validated and standardized protocols, highlighting issues such as errors in dose scaling from in vitro to in vivo models, inaccuracies in tumor models, variations in receptor expression, and the non‐standardization of animal model selection, which often leaves the decision to individual researchers, among other issues.^63^ Differences between preclinical and clinical outcomes could be due to different receptor expressions affecting absorption if there is receptor‐mediated uptake, distribution if there are tissue‐specific receptor targets, excretion in the case of renal filtration receptors, and most notably, metabolism of nanoparticles capable of being metabolized (e.g., polymeric, lipid, and protein nanoparticles). It has been recommended that two preclinical models, long‐term studies, and multiple doses should be explored to increase the feasibility of translation in nanomedicine.^91^

#### 3.1.5 Quantifying the impact of non‐specific nanoparticle effects

Unintended off‐site and off‐target effects of nanoparticles remain a concern for clinical translation. The likelihood that a nanoparticle finds its target site is often overstated and it has been established that 95% of administered nanoparticle doses are sequestered by filtration organs (liver, spleen, and lungs) and never reach their intended targets.^63^, ^86^ Nanoparticle accumulation in organs responsible for clearance (liver, spleen, kidney, etc.) initiates immune responses,^92^ which can also result in unforeseen toxicities. There is growing evidence that an administered dose can overwhelm the MPS, rendering it unable to respond to pathogens.^93^ Strategies to reduce MPS recognition can also result in non‐specific effects. As introduced in Section 3.1.2, PEGylation alters the PK by decreasing renal filtration due to its contribution to molecular weight, as well as protecting from proteolytic degradation.^7^ In rare cases, PEGylated nanoparticles can result in off‐target anaphylactic events as well as immune responses due to anti‐PEG antibodies (mainly IgG and IgM Abs).^71^, ^94^ It has also been established that the common use of PEG‐based excipients increases the expression of anti‐PEG antibodies that can increase the clearance of PEGylated nanoparticles.^95^ Recurrent administration of PEGylated nanoparticles can also increase their clearance.^96^ For example, LNPs administered to mice with pre‐existing anti‐PEG antibodies showed a lower AUC, reduced biodistribution to the tumor target, and accelerated clearance.^97^ It has been recommended that anti‐PEG antibody screening be conducted before administration of formulations with PEG.^98^ Other polymers have been explored as alternatives to PEG.^99^ A comprehensive understanding of the interactions between nanoparticles and biological systems is essential to minimize off‐target effects and enhance the safety profile of these therapies.

#### 3.1.6 Long‐term biodistribution and PK studies

In addition to acute studies, long‐term biodistribution and clearance studies should be conducted in order to predict toxicity due to organ accumulation, low clearance, or high systemic exposure.^100^ Chronic and sub‐chronic studies are sparse and should be a focus of future studies.^93^, ^100^ However, there are significant issues in collecting long‐term data, including temporal and financial restraints associated with long‐term studies, shortened lifespans for inbred or immune‐deficient laboratory animals, temporally dependent tissue collection that requires a large number of animals, and detection sensitivity of the low amount of nanoparticles. In such cases, PK modeling can be employed to predict PK and associated toxicity and inform appropriate dose recommendations.^101^, ^102^ To best understand potential toxicity, it is also essential to compare the toxicity profile of a nanoparticle that is not being administered IV to an IV administration, as the non‐IV administration should show lower AUC, bioavailability (*F*), and *C*_max_ in plasma. Further, the effect toxicity might have on PK will be different after single and multiple doses; therefore, the most thorough route of toxicity exploration would be to conduct PK studies with both. Clinically, it is common to see multiple doses administered over long term scales, but preclinically, often single doses are analyzed, particularly for studies conducted over a shorter time scale. PK modeling can be used to simulate a different dosing schedule, although having a dataset to validate the model would give the model more predictive power. Multiple doses will affect PK through accumulation in tissues, saturation of clearance or target pathways, and changes in distribution or metabolism. These effects can lead to nonlinear PK and multiple absorption phases, and increased toxicity due to prolonged exposure or immune activation. Evaluating repeat dosing more reflective of a clinical regime is crucial to understanding long‐term behavior, ensuring predictable therapeutic outcomes, and minimizing potential adverse effects.

Understanding the fate, retention, and clearance of nanoparticles is critical for toxicity studies because these PK properties directly determine the extent and duration of exposure to both target and off‐target tissues. For example, nanoparticles with prolonged retention in non‐target organs may lead to chronic toxicity or organ‐specific damage. Similarly, inefficient clearance or accumulation in sensitive tissues such as the kidneys, brain, or lungs can exacerbate inflammation, oxidative stress, or other adverse effects. Retention studies help identify potential areas where nanoparticles might accumulate, enabling researchers to assess the risk of sustained exposure. Studies determining nanoparticle fate are crucial for tracking nanoparticle degradation and production of potential toxic byproducts. Clearance studies provide insights into the elimination pathways ensuring that nanoparticles and their metabolites are efficiently excreted without causing long‐term toxicity or accumulation. Currently, long‐term studies that quantify fate, retention, and clearance are deficient in the nanomedicine field.

### 3.2 Scale‐up and manufacturing

Many promising nanoparticle formulations fail to reach commercialization due to the challenges associated with manufacturing these complex systems on a large scale, regulatory uncertainties, and market competition. Developing controllable, reproducible, and scalable formulation technologies is a considerable challenge.^103^ The scale‐up of formulation requires methods to manufacture nanoparticles with stable physicochemical properties and minimal batch‐to‐batch variations.^104^ Translating laboratory‐scale production to commercial levels while maintaining the quality, uniformity, and cost‐effectiveness of the nanoparticles poses hurdles in achieving manufacturing scalability. Platforms demanding intricate or labor‐intensive synthesis methods often face constraints in terms of large‐scale pharmaceutical manufacturing, limiting their potential for effective clinical translation.^105^, ^106^, ^107^, ^108^ Simplifying nanoparticle design is necessary for reproducible large‐scale manufacturing.^109^ An example of this is the Moderna COVID‐19 vaccine, which uses cationic lipids within the LNP to stabilize the mRNA, cholesterol to enhance the fusion with the cell membrane and to maintain the fluidity of the LNP, PEG to sterically protect the LNP, and a zwitterionic phospholipid to form a bilayer. The simplicity of the LNP design combined with the robust understanding of their individual properties streamlined the manufacturing process. Moreover, the global production of 19 million COVID‐19 vaccine doses within a matter of months,^9^ and 807 million doses distributed by Moderna alone in 2021,^110^ demonstrated the scalability of this platform, highlighting how well‐defined, proven components can expedite manufacturing at an unprecedented scale.

In addition to scaling nanoparticle formulation, maintaining the stability and shelf‐life is critical for nanoparticle use in the clinic. Factors such as aggregation, degradation, or changes in physicochemical properties over time can affect the efficacy and safety of the nanoparticles. Developing stable formulations that can be stored for extended periods is essential for successful translation. According to the International Council for Harmonization of Technical Requirements for Pharmaceuticals for Human Use (ICH) guidelines, for a drug product (including nanoparticles), long‐term stability studies up to 12 months are recommended, and a purity of ~95% is desired.^111^ Further, quality by design (QbD) procedures, as recommended by the ICH, have increased the success of nanoformulations.^112^, ^113^, ^114^, ^115^ For example, recent analysis has shown hydrolytic degradation of PEGylated and polymer‐based colloidal nanoparticles in the long term undermines the nanoparticle shelf‐life.^116^ Currently, the gold standard in drying colloidal nanoparticles is lyophilization, which can introduce additional formulation components that impact the stability of particle size and incorporated active agent.^117^ While advancements in formulation techniques and manufacturing processes have enabled more scalable and efficient nanoparticle production, these same design elements critically impact PK.

Through control of nanoparticle physicochemical properties and manufacturing for shelf‐life stability, nanoparticle development aims to minimize adverse events while providing a therapeutic level of active agent to the patient. PK modeling is essential to understanding and predicting this balance.

## 4 PK MODELING FOR TRANSLATION OF NANOPARTICLE‐BASED THERAPEUTICS

**FIGURE 3:**
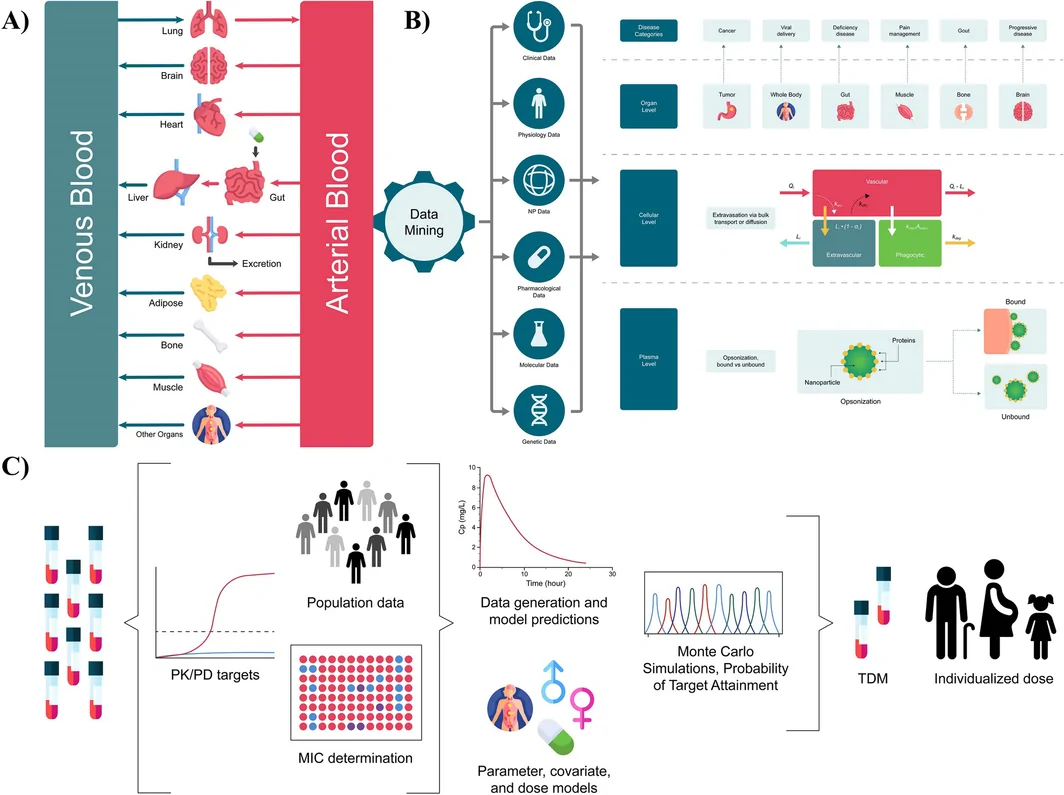
The workflow of A) PBPK modeling, B) PopPK modeling, C) Quantitative Systems Pharmacology (QSP) modeling. Panel C is adapted from https://www.cosbi.eu/case‐studies/developing‐a‐unified‐qsp‐platform‐for‐lysosomal‐storage‐disorders‐lsds.^118^

PK modeling is a critical but underutilized tool that can assess the translational potential of nanoparticles by predicting their ADME and the active agent payload. The most common PK modeling methods, mechanistic modeling (quantitative systems pharmacology (QSP)), PBPK, and population PK (PopPK), are shown schematically in Figure 3. PK modeling generally employs compartmental and non‐compartmental approaches. Compartmental models, such as PBPK, PopPK, and QSP models, divide the body into compartments to describe active agent distribution and dynamics using differential equations. This method, which comprises multiple compartments representing different physiological spaces, is particularly suited to nanoparticle modeling due to its unique distribution properties. Non‐compartmental modeling, by contrast, simplifies the analysis by focusing directly on active agent concentration‐time data and calculating parameters like AUC, CL, volume of distribution (*V*_d_), half‐life (*t*_1/2_), and *F* without compartmental assumptions. While compartmental models provide detailed insights but require more data and assumptions, non‐compartmental models offer quicker, practical assessments with less data. This is particularly useful for early‐phase active agent development, where simplicity is preferred. Both modeling approaches are valuable tools in PK analysis, and the choice between them depends on the specific objectives of the study and the available data.

To address some of the challenges discussed in Section 3.2 specifically, modeling could assist in manufacturing inconsistencies for nanoparticles. Machine learning models can optimize manufacturing parameters based on data and experimental results known a priori.^119^, ^120^, ^121^ Monte Carlo methods have been used to model and predict the effects of various parameters on nanoparticle properties, such as size and surface characteristics, to identify parameters that affect nanoparticle quality.^122^ Computational fluid dynamics models help in understanding the flow dynamics, mixing, and reaction conditions in nanoparticle synthesis reactors, which can lead to more efficient production and better control over particle size and distribution.^123^ Discrete element modeling can be employed to simulate the behavior of particles in milling and grinding processes, which are crucial for nanoparticle production.^124^

PK modeling can assist in the translation of nanoparticles by predicting biodistribution, predicting release kinetics, accounting for variability in patient response, predicting interactions with biological barriers, assessing long‐term toxicity and clearance, and enhancing clinical trial design.

### 4.1 Mechanistic models

PK modeling of nanoparticles is complex due to their unique characteristics as well as a lack of characterization of complex absorption processes. Nanoparticle absorption can only be measured with oral, rectal, nasal, sublingual/buccal, ocular, transdermal, and subcutaneous administration, but nanoparticles encounter complex physiological barriers that small molecules do not, regardless of administration route.^84^ New approach methodologies, also referred to as next‐generation risk assessment, such as in vitro‐in vivo extrapolation (IVIVE), can provide valuable support to nanoparticle translation and PK modeling. IVIVE is the process of correlating in vitro patterns with in vivo effects. The active agent concentration in an in vitro experiment can be scaled up to physiologically relevant concentrations using PBPK modeling to predict adverse events, therapeutic doses, and efficacy in humans.^125^ IVIVE can be used to improve PK models of nanoparticles by providing a more comprehensive picture of what nanoparticle characteristics affect model parameters. To create a mechanistic model, Quantitative Systems Pharmacology (QSP) models combine quantitative data from various sources, such as preclinical experiments, clinical trials, and in vitro studies.^20^ QSP models aim to understand the underlying biological mechanisms/pathways to provide insights into how an active agent (or nanoparticle) works and potential off‐target effects. The United Kingdom National Research Council (NRC) has published reports on how the combined use of in vitro high‐throughput screening and computational models can be used to replace traditional animal models.^126^, ^127^, ^128^

Tailoring PK models to specific disease conditions, considering altered physiological parameters or pathological changes, is crucial. Integrating imaging data, such as positron emission tomography (PET) or magnetic resonance imaging (MRI), validates and refines PK models by providing real‐time information on nanoparticle distribution in vivo. For example, using fluorescence microscopy, kinetic models for length‐fractionated single‐walled carbon nanotubes (SWNT) and AuNPs were designed to correlate endocytosis rate with size/geometry developed by Jin et al.^129^ In another instance, the Enhanced Permeability and Retention (EPR) effect was studied a priori with a meta‐analysis of delivery to solid tumors; findings were then applied to the author's recent AuNP study to develop a new PBPK model.^130^ Cell‐specific interactions can also be incorporated into models. Membrane receptors can bind nanoparticles; therefore, the rate of receptor recycling, nanoparticle adsorption to the surface, and nanoparticle internalization are essential. Disease‐specific modeling tailors PK models to specific disease conditions, accounting for altered physiological parameters or pathological changes that may impact nanoparticle PK. With the plethora of clinical trial data for nanoparticle use (successful or unsuccessful) (Tables 1, 2, 3, Table S1), researchers can refine and validate PK models based on feedback from clinical trials, incorporating real‐world data to improve the accuracy of predictions and optimize therapeutic strategies.

Bayesian analysis provides a powerful framework for evaluating models, estimating parameters, and quantifying uncertainty. The combination of prior knowledge with observed data can enhance inference and decision‐making. Bayesian population analysis has been demonstrated for nanoparticle use in rat models^131^, ^132^, ^133^, ^134^, ^135^, ^136^ and has also been used for in vitro experiments of nanoparticles.^137^, ^138^, ^139^ For example, Schmidt et al. tested parameter–covariate relationships of camptothecin nanoparticles in their PopPK model based on human data.^140^ Bayesian techniques facilitate the identification of model inadequacies and parameter‐identifiability issues, improving the model's predictive accuracy and reliability. Bayesian population analysis utilizing Markov chain Monte Carlo (MCMC) simulation can be conducted to derive parameter distributions instead of singular point estimates for subsequent analyses of uncertainty and variability.^141^ Furthermore, Bayesian analysis enables the exploration of alternative model structures and assumptions, facilitating model refinement and hypothesis testing. Overall, Bayesian evaluation of models enhances the transparency, rigor, and utility of PK modeling in active agent development and regulatory decision‐making processes.

Stochastic modeling of nanoparticles employs probabilistic and statistical methods to capture the inherent variability and uncertainty in their behavior within biological systems. Unlike deterministic modeling, which provides a single set of predictions based on fixed parameters, stochastic models acknowledge the dynamic and probabilistic nature of interactions in complex environments.^142^ In stochastic modeling, various sources of variability, such as differences in heterogeneous biological responses and fluctuating physiological conditions, are represented as probability distributions.^142^ Monte Carlo simulations are often used in stochastic modeling, where multiple simulations are run with randomly sampled input parameters to generate a range of possible outcomes.^143^ This approach is particularly relevant for nanoparticles due to their diverse physicochemical properties, potential for complex interactions with biological components, and sensitivity to microenvironmental variations. Stochastic modeling enables the exploration of a spectrum of scenarios, providing insights into the range of possible nanoparticle behaviors under different conditions. Applications of stochastic modeling for nanoparticles include predicting variations in biodistribution, assessing the impact of stochastic cellular interactions, estimating the likelihood of different PK outcomes, and for use in combination with PopPK modeling.^144^, ^145^, ^146^, ^147^

#### 4.1.1 Limitations

Mechanistic modeling, while powerful, is confronted with several critical issues that necessitate thorough investigation. Once the model structure is determined, it is essential to select which parameters can be identified a priori from the existing literature. The remaining parameters can subsequently be estimated from empirical data. Obtaining a highly accurate estimation of mechanistic model parameters from empirical data is often challenging due to the intricate nature of biological systems and the limited availability of precise measurement methodologies, leading to uncertainty in model predictions as inaccuracies in parameters. Furthermore, the complexity inherent to in vivo biological systems further complicates models, especially when considering the intrinsic variability among sub‐populations due to genetic and physiological factors, such as those with specific organ deficiencies.^148^ PK modeling, particularly in cancer research and inter‐individual differences of tumor type, has occasionally led to inaccurate predictions when scaled from preclinical to clinical models.^49^ Mechanistic models often oversimplify these complex interactions, resulting in predictions that deviate from actual outcomes. While models are valuable tools for prediction and understanding, they are inherently based on assumptions and abstractions and therefore cannot perfectly represent real‐world data.

Bayesian analysis can be computationally complex and time consuming. Ensuring convergence of MCMC simulation due to autocorrelation or multimodality can lead to unreliable parameter estimates. Additionally, running multiple chains from different starting points is standard practice, and if they converge to different regions, it signals non‐convergence. To avoid these issues, scientists can improve model parameterization, use a priori information, carefully choose initial values, run multiple well‐tuned chains, and apply convergence diagnostics to ensure adequate sampling.

### 4.2 PBPK modeling

PBPK modeling integrates physiological parameters with active‐agent‐specific characteristics to predict active‐agent concentrations in different tissues over time. Traditional compartmental modeling is adapted to consider various compartments such as blood, tissues, and organs. Sub‐compartments are often included and may be physiological spaces like interstitial spaces, vascular endothelium, tissue layers, macrophages, kidney nephrons, or tumor microenvironments. Critical elements of PBPK modeling include organ‐specific blood flow, tissue composition, and physicochemical properties of the active agent. This approach allows for a more mechanistic understanding of active agent behavior within the body. PBPK modeling allows for relatively facile scaling, which can be useful for interspecies scaling to make predictions in a new population. Allometric scaling is the most comprehensive method for interspecies scaling and is applied to organ weights, blood perfusion rate, hepatocytes, or renal cells to account for metabolic activity and mass transfer area.^149^

PBPK modeling of nanoparticles is unique in that it considers physiological parameters and nanoparticle‐specific characteristics. Developing PK models for nanoparticles involves employing specialized strategies that integrate information on nanoparticle physicochemical properties, surface modifications, Hill coefficient, phagocytic uptake and release, and interactions with biological components to simulate their biodistribution and fate within the body. This allows for a mechanistic approach to determine the most influential parameters on the model and predict active agent disposition in clinical applications. In addition, challenges in translation such as lack of absorption knowledge, intricate structure, extravasation, inter‐species scaling, corona formation/protein binding, tissue binding, EPR, phagocytosis, and biodistribution can be modeled. For example, PBPK modeling has been applied to compare the PK of paclitaxel formulated in different vehicles, including photosensitive paclitaxel prodrug,^150^ interspecies predictions,^151^ poly(lactic‐co‐glycolic acid)‐bound nanoparticles,^152^ and albumin‐bound nanoparticles.^153^ The model considered factors such as solubility, particle size, and tissue distribution to predict the impact of formulation on drug exposure, which guided formulation optimization for enhanced therapeutic efficacy of a drug formulation. Nanoparticle PBPK modeling differs from small molecule modeling by incorporating particle‐specific processes such as size‐dependent tissue uptake, opsonization, and clearance via the MPS, which are not typically relevant for small molecules. Additionally, nanoparticles may undergo dissolution, aggregation, or surface modifications in vivo, requiring dynamic modeling of physicochemical transformations that are less significant for small‐molecule drugs.

Current PBPK models of nanoparticles are summarized in Table 4. PBPK model advancements for nanoparticles, including artificial intelligence and machine learning‐assisted modeling, have been recently reviewed.^154^, ^155^ PBPK models are verified with datasets and can be updated based on new datasets for further validation. Validation is confirmed using average fold errors (AFE) or absolute average fold errors (AAFE), the predicted versus observed values ratio. Generally, it is desirable to have less than a 2.0‐fold error to maintain confidence in the predictive power of the model. While physiological parameters like blood flow rates and organ size can be found in the literature, nanoparticle‐specific parameters will require in vitro testing and optimization in the model. Local and global sensitivity analysis (SA) techniques can be employed. Using local SA, the degree of parameter influence can be determined using the relative change in the AUC after a 1% change in each parameter (p).^156^, ^157^ Global SA techniques have been described elsewhere.^158^, ^159^, ^160^ Dual PBPK nanoparticle and active pharmaceutical ingredient models can be employed when the nanoparticles are used as an active agent carrier. Previously developed nanoparticle PBPK models explore biodistribution,^50^, ^129^, ^131^, ^134^, ^141^, ^161^, ^162^, ^163^, ^164^, ^165^, ^166^, ^167^, ^168^, ^169^, ^170^, ^171^, ^172^, ^173^, ^174^, ^175^, ^176^, ^177^, ^178^, ^179^, ^180^, ^181^, ^182^, ^183^, ^184^ toxicity,^156^, ^185^, ^186^ efficacy,^187^, ^188^, ^189^, ^190^, ^191^ multi‐route modeling,^136^ design maps,^189^, ^192^, ^193^ or PK of new formulations.^189^, ^194^, ^195^

**TABLE 4:** PBPK models of nanoparticles.

Model	Model characteristics				Biological focus		Software used
Author	NP type	# Compartments	Organ compartments	Clearance	Species	Route	Software tools
W. Chou, et al.	AuNPs	7 organs (9 Inc. Blood)	Upper airway, tracheobronchial, spleen, liver, GI tract, kidney, remaining tissue, blood (arterial and venous)	“Feces and Urine”	Rats	IV, OG, IT, EI	R‐Shiny, Berkeley Madonna
M. Aborig, et al.	AuNPs	15 organs (17 Inc. Blood)	Heart, kidneys, muscle, skin, brain, adipose, gonads, liver, stomach, spleen, pancreas, small intestine, large intestine, bone, lungs, blood (arterial and venous)	“Excretion”	Mice, Rats	IP	MATLAB, IQMTools
G. Bachler, et al.	AgNPs and Ag Ions	11 organs (12 Inc. Blood)	Liver, lungs, spleen, brain, kidneys, heart, intestines, muscles, skin, bone marrow, testes, blood	“Biliary, Direct, and Urinary”	Humans, Rats	PO, Dermal, INH, IV	NA
G. Bachler, et al.	Nano‐TiO2	11 organs (12 Inc. Blood)	Brain, skin, lung, stomach, heart, intestine, bone, liver, spleen, kidneys, remainder, blood	“Biliary, Direct, and Urinary”	Humans, Rats	PO, Dermal, IV	NA
W. Chen, et al.	Nano‐ZnO_2_	8 organs (10 Inc. Blood)	Lung, heart, spleen, liver, GI, brain, kidney, carcass, blood (arterial and venous)	Parameters “k_GI, k_Li, k_ki”	Mice	IV	Crystal Ball, TableCurve 2D
MJ. Gilkey, et al.	Dexamethasone (Dex)	4 organs (5 Inc. Blood)	Liver, spleen, kidney, other, blood (plasma)	“Urine”	Mice	IV	MATLAB
Y. Cheng, et al.	AuNPs	8 (healthy), 9 (tumor burdened), 11 (Inc. blood)	Lung, liver, spleen, kidney, brain, muscle, remaining, tumor, blood (arterial and venous)	“Feces and Urine”	Mice	IV	Berkeley Madonna
U. Carlander, et al.	Nano‐CeO_2_	8 organs (10 Inc. blood)	Lung, bone marrow, brain, heart, kidney, other, liver, spleen, blood (arterial and venous)	“Feces and Urine”	Rats	INH, IV, IT, PO	acslX Libero, Berkeley Madonna
A. Péry, et al.	C (w/ Tc‐99 m)	22 organs (24 Inc. blood)	Lungs, airways, stomach, brain, heart, fat, marrow, muscles, adrenals, skin, thyroid, breast, kidneys, others, urine, pancreas, spleen, stomach lumen, gut, testes, liver, gut lumen, blood (arterial and venous)	“Urine”	Humans	INH	MCSim
B. Howell, et al.	Liposomes	13 organs (15 Inc. blood)	Lungs, adipose, brain, heart, liver, pancreas, gut, spleen, thymus, bone, skin, muscle, kidney, blood (arterial and venous)	“Hepatic and Renal”	Humans	PO, IV, Epidural, IP, IC	MATLAB
R. Rajith, et al.	Nano formulations	18 organs (22 Inc. blood)	Adipose, bones, brain, muscle, skin, gonads, thymus, lungs, IM depot, pancreas, spleen, stomach, liver, kidneys, small intestine, metabolism, elimination, blood (portal vein, veins, arteries, capillaries)	“Metabolism and Elimination”	Humans	IM	MATLAB
F. Jung, et al.	Polycaprolactone	2 (in vitro)	Unstirred water layer, intestinal barrier	NA	In Vitro	NA	Phoenix WinNonlin
X. Lu, et al.	Liposomes	9 organs (11 Inc. blood)	Lung, brain, heart, spleen, liver, gut, kidney, muscle, remainder, blood (artery and venous)	“Liver and GI”	Mice, Rats, Humans	IV	MATLAB
D. Li, et al.	Polyacrylamide nanoparticles	8 organs (10 Inc. blood)	Lungs, spleen, liver, kidneys, heart, brain, bone marrow, rest of the body (skin, muscle, skeleton), blood (arterial and venous)	“Excreta”	Rats	NA	Berkeley Madonna, acslX
M. Li, et al.	PLGA	1 (intracellular)	MCF7 cell line	NA	In vitro	PBS	Origin
M. Li, et al.	PLGA	8 organs (10 Inc. blood)	Lungs, GI tract, GI lumen, liver, urine, spleen, kidney, body, blood (artery and venous)	“Urine”	Mice	IV	MATLAB
M. Li, et al.	PTX Prodrug	8 organs (9 Inc. tumor) (10 Inc. blood)	blood (Inc. plasma), liver, spleen, GI tract, muscle, skin, kidney, remainder, tumor	“Hepatic and Renal”	Mice	IV	ADAPT, Berkeley Madonna
D. Li, et al.	Nano‐CeO_2_	8 organs (10 Inc. blood)	Brain, other, heart, kidney, liver, spleen, GI tract, blood (Inc. artery, venous), airway	“Excretion”	Rat	INH	Berkeley Madonna
D. Dong, et al.	SNX‐2112 Nanocrystals	7 organs (8 Inc. blood)	Lung, blood (plasma), remainder, heart, kidney, spleen, liver, intestine	“Hepatic and Renal Uptake”	Rat	IV	MATLAB
L. Sweeny, et al.	Metal NPs	13 organs (15 Inc. blood)	Olfactory region, upper airways, alveolar region, interstitium, lymph nodes, gut contents, GI tissue, liver, spleen, kidney, heart, brain, others, blood (arterial and venous)	“Urine and Feces”	Rat	INH	MCSim
D.P.K. Lankveld, et al.	AgNPs	4 organs (5. Inc. blood)	Blood, remaining, liver, spleen, kidney	Parameters “k_k,x”	Rat	IV	ACSL
L. Kagan, et al.	AmBisome	7 (8 Inc. blood)	Lung, blood (arterial and venous plasma), heart, kidney, gi tract, liver, spleen, remainder	“Biliary and Urinary”	Mouse, Rat	IV	MATLAB
Z. Lin, et al.	AuNPs	7 (9. Inc. blood)	Lung, remainder, brain, spleen, liver, kidney, blood (arterial and venous plasma)	“Liver and Gut”	Mice	IV	NA
Z. Mager, et al.	Au/Dendrimer CNDs	8 (9 Inc. blood)	Blood, lung, heart, kidney, muscle, brain, spleen, liver, other	“Urine” and “Feces”	Mice	IV	Berkeley Madonna
P. Dogra, et al.	SiO_2_	11 (12 Inc. blood)	Brain, heart, lungs, blood (plasma), liver, kidneys, spleen, gi tract, muscle, tumor, others, lymph nodes	“Excretia”	Rats	IV	MATLAB
R. Kumar, et al.	ORMOSIL	8 (9. Inc. blood)	Liver, lungs, kidney, spleen, stomach, intestine, skin, heart, blood	“Feces and Urine”	Mice	IV	NA
J. Hillyer, et al.	Colloidal Au	8 (9 Inc. blood)	Blood, brain, lung, heart, kidney, spleen, liver, small intestine, stomach	NA	Mice	PO	NA
A. Klapproth, et al.	SPIONs	12, 13 (Inc. tumor), 14 (Inc. blood)	Bone, spleen, kidney, heart, lung, muscle, brain, colon, intestine, liver, stomach, skin, tumor, blood	NA	Mice	IV and i.t.	MATLAB
A. A. Antsiferova, et al.	Ag	6, 7 (Inc. Blood)	Brain, lungs, liver, testes, gastrointestinal tract (GIT), exposure site, blood	“Environmental (Feces and Urine)”	Mice	PO	SciPy (Python)
E. O. Kutaumova, et al.	Albumin	5, 7 (Inc. blood)	Arterial plasma, venous plasma, lungs, spleen, liver, kidneys, remainder	Bile and Urine	Mice	IV	BioUML
H. Parhiz et al.	LNPs	7, 8 (Inc. blood)	Venous blood, lungs, heart, kidneys, spleen, liver, remainder (accounting for portal vein organs), all other tissues	NA	Mice	IV	ADAPT5
I. Gosens et al.	Cerium dioxide (CeO_2_), titanium dioxide (TiO_2_)	6, 7 (Inc. blood)	Lung, mediastinal lymph nodes, liver, spleen, kidney, blood, excreta	Urine	Rat	INH	NA

Abbreviations: CNDs, composite nanodevices; Dex, dexamethasone‐encapsulated; dermal, dermal; EI, endotracheal inhalation; epidural, epidural; IC, intracardiac; IM, intramuscular; INH, inhalation; IP, intraperitoneal; IT, intrathecal; i.t., intratumoral; IV, intravenous; NA, not applicable/not available; OG, orogastric; PO, oral; PBS, phosphate buffered saline; PLGA, poly(lactic‐co‐glycolic acid).

A notable example is the work of Dogra et al., who developed a multi‐scale PBPK model that includes a tumor compartment.^190^ This model, encompassing 12 key tissue compartments, is designed to examine the influence of nanoparticle properties, tumor‐specific variables, and individual physiological differences on factors such as plasma circulation time, MPS accumulation, excretion kinetics, and tumor delivery efficiency in rats. Given the multiscale nature of this PBPK model, organs are subdivided into intravascular, extravascular, and phagocytic sub‐compartments, incorporating sub‐cellular dynamics like vessel margination and cell‐scale phenomena such as phagocytosis. Through both local sensitivity analysis and global sensitivity analysis, the study highlighted the importance of several factors in influencing delivery to the tumor interstitium, which include nanoparticle degradation rate, tumor blood viscosity, nanoparticle size, tumor vascular fraction, and tumor vascular porosity. This model was not scaled to humans and is only applicable to rats.

Another model of note is an AuNP mouse PBPK model created by Aborig et al. that was extrapolated to rats.^161^ This model includes long‐term modeling up to 56 days and 15 organ compartments separated into four sub‐compartments: plasma, vascular endothelium, macrophages, and interstitial space. Parameter optimization was completed through local sensitivity analysis and parameter correlation. The extrapolated rat PBPK model indicated that the model was accurate based on AFE and demonstrates the important concept of interspecies extrapolation, which is critical in translational research. While the model was scaled between rodent species, there is no human extrapolation.

#### 4.2.1 Limitations

Nanoparticle PBPK models are limited by scarce in vivo data, especially in humans, and an incomplete understanding of nanoparticle‐biological interactions such as protein corona formation, aggregation, and MPS uptake and clearance. Variability in nanoparticle properties and in vivo size/hydrodynamic radius further complicate accurate modeling. These limitations reduce confidence in translating animal data to humans, leading to uncertainty in exposure predictions, dose selection, and safety assessments. To address these challenges, future models should integrate IVIVE approaches, dynamic physicochemical processes, and species‐specific MPS scaling, supported by standardized experimental data and machine learning techniques to manage uncertainty.

### 4.3 PopPK modeling

PopPK focuses on understanding variability in active agent PK across individuals by considering factors such as age, weight, genetic variations, and disease status. Utilizing nonlinear mixed‐effects models, PopPK predicts active agent concentrations in large, heterogeneous groups without the need for dense individual sampling, making it particularly advantageous in clinical settings. Unlike the analysis of single‐subject data, PopPK does not necessitate rich data (many observations per subject) or structured sampling time schedules. Instead, it can utilize sparse data (few observations per subject) or a combination thereof. This approach facilitates the optimization of dosing strategies and the prediction of variability in efficacy and toxicity, thereby enhancing the translation of nanoparticles from early‐phase trials (Phase I) to later stages (Phase III). Common PopPK modeling approaches for nanoparticles include multi‐compartment modeling, log‐normal distribution of observations, linear kinetics and elimination, and proportional residual error models for addressing variability in measurements and predictions.

Current PopPK models of nanoparticles are summarized in Table 5. PopPK models have been extensively used in various nanoparticle applications, including formulation studies,^196^, ^197^ cancer therapeutics,^140^, ^198^, ^199^, ^200^, ^201^, ^202^ imaging,^203^ and PK studies.^204^, ^205^ For instance, K. Schmidt et al., developed a PopPK model for NLG207, a nanoparticle‐drug conjugate of camptothecin (CPT), aimed at improving its delivery and PK properties.^120^ Utilizing data from 27 patients across two Phase II trials, the study employs NONMEM software for nonlinear mixed‐effects modeling to establish two‐compartment models for both conjugated and free forms of CPT, adjusted for body size via allometric scaling. The analysis reveals differentiated PK behaviors between the drug forms, highlighted by an initial rapid clearance of CPT from the nanoparticles, transitioning to a slower, steady‐state release. This detailed modeling provides mechanistic insights into CPT dynamics, aiding in optimizing dosing strategies and predicting patient‐specific responses, thereby enhancing the clinical utility of NLG207.

**TABLE 5:** PopPK models of nanoparticles.

Model identification	Model characteristics		Biological focus		Software used
Author	Type of NP	# Compartments	Species	Administration route	Software tools
A. Funguetto‐Ribeiro, et al.	Polymeric nanocarriers (NCs with polysorbate 80, PEG, and Chitosan)	2	Rats	IV, IP	MonolixSuite, Simulation Plus
H. Wu, et al.	PEGylated Liposomal CPT‐11	2	Humans	IV	NONMEM, S‐PLUS
S‐H. Jeong et al.	Poly Lactic‐co‐Glycolic Acid (PLGA)	1	Mice	IV, OG	Phoenix NLME
H. Zhou, et al.	Liposome	3	Humans	IV	NONMEM
E. Yaghini, et al.	Quantum Dots (PEGylated Bio CFQD NPs)	3	Mice	IV	NONMEM
K. Schmidt, et al.	NLG207 (Beta Cyclodextrin and Polyethylene Glycol)	4	Humans	IV	NONMEM
G. Hempel, et al.	Liposome	1	Humans	IV	NONMEM
K. E. Stott, et al.	Liposome	2 and 3	Humans	IV	Pmetrics (R)
Y. Li, et al.	Nanoparticle albumin‐bound paclitaxel	3	Humans	IV	NONMEM
B. S. Adiwijaya	Liposome	2	Humans	IV	NONMEM
M. A. Amantea	Liposome	2	Humans	IV	IT2S

Abbreviations: CNDs, composite nanodevices; dex, dexamethasone‐encapsulated; dermal, dermal, EI, endotracheal inhalation; epidural, epidural; IC, intracardiac; IM, intramuscular; INH, inhalation; IP, intraperitoneal; IT, intrathecal; i.t., intratumoral; IV, intravenous; NA, not applicable; OG, orogastric; PO, oral; PBS, phosphate buffered saline; PLGA, poly(DL‐lactide‐co‐glycolide).

PopPK allows for fixed effects analysis that utilizes the average PK parameters in the population to describe trends in the data, causing inter‐individual variability. Random effects account for variability between individuals, not explained by measured covariates. They capture the extent to which an individual's PK parameters deviate from the population average, reflecting natural variation. Moreover, PopPK modeling robustly incorporates various biological and demographic covariates such as ethnicity, age, weight, genetic markers, and metabolic biomarkers, like bilirubin, creatinine, glomerular filtration rate, aspartate transaminase, and alanine transaminase, to refine predictions and personalize therapy. Inter‐subject variability can also result from differences in administration times/doses instead of from covariates.^205^ Model goodness‐of‐fit evaluation can be completed with Akaike's information criterion,^202^, ^205^ ensuring robust predictions.

Several FDA‐approved nanoparticle formulations have been subjected to PopPK analysis,^200^, ^201^, ^202^, ^204^, ^205^ demonstrating the approach's utility in guiding therapeutic development and enhancing the precision of nanoparticle‐based treatments. For instance, the study by Y. Li et al. exemplifies the application of FDA‐approved PopPK models in a pediatric setting, where the PK of nanoparticle albumin‐bound (nab)‐paclitaxel was investigated.^180^ By employing a three‐compartment model with saturable elimination, adjusted for patient‐specific factors such as body size and maturation, this research not only supports regulatory requirements but also aids clinical decision‐making, showcasing the integration of PopPK modeling in active agent development and precision medicine, especially in addressing the pharmacological challenges in treating cancers with nanoparticle‐based therapies.

#### 4.3.1 Limitations

Similar to PBPK modeling, PopPK modeling of nanoparticles is challenged by their complex and variable in vivo behavior. High inter‐individual variability in MPS clearance, immune response, and organ function can lead to significant differences in nanoparticle distribution and clearance. Limited clinical PK data, due to sparse sampling and analytical difficulties in quantifying nanoparticles in biological matrices, further constrains model robustness. Additionally, nanoparticles often undergo dynamic physicochemical changes such as aggregation, dissolution, or protein corona formation, which are not easily captured in conventional PK frameworks. Their disposition frequently exhibits non‐linear or time‐dependent kinetics due to saturable cellular uptake and tissue‐specific retention. Finally, the lack of standardized assays and batch‐to‐batch variability in nanoparticle formulations complicates both data interpretation and population‐level predictions, underscoring the need for advanced modeling strategies.

## 5 CONCLUSIONS

In the convergence of nanomedicine and gene therapy, the success of lipid nanoparticles in COVID‐19 mRNA vaccine delivery has highlighted the potential of nanoparticles as carriers for genetic payloads. Another crucial advancement lies in the development of mechanistic models that integrate cellular pathways, immune responses, and disease mechanisms to refine nanoparticle design and predict therapeutic outcomes.

Despite these promising advancements, barriers must be addressed to fully realize the potential of the proposed MIND workflow. One of the most pressing issues is the lack of standardized methodologies for nanoparticle characterization and PK data collection, though the Nanotechnology Characterization Lab has made advances.^206^, ^207^, ^208^, ^209^ Variability in nanoparticle synthesis, characterization techniques, and bioanalytical methods has led to inconsistencies in preclinical and clinical data, making it difficult to compare and validate PK models across different studies.

While model‐informed drug development (MIDD) is widely used by the pharmaceutical industry and is recommended by the FDA for traditional pharmaceuticals,^210^ regulatory agencies and the pharmaceutical industry have been slower to embrace modeling strategies for nanomedicine.

Nanoparticles have demonstrated significant potential in enhancing drug stability and solubility, as well as enhancing targeted delivery; yet their clinical translation has been hindered by off‐target effects, manufacturing complexities, and the lack of robust PK data, as analyzed in this review.

One of the key frontiers in MIND is the refinement of PBPK models to enhance interspecies translation. In addition to PBPK models, PopPK modeling will play a critical role in optimizing dosing strategies and accounting for inter‐individual variability. Given the significant variability in how nanoparticles behave in different patient populations due to factors such as age, disease state, and immune function, PopPK models must be expanded to incorporate covariates.

## 6 AUTHORS' PERSPECTIVE

**FIGURE 4:**
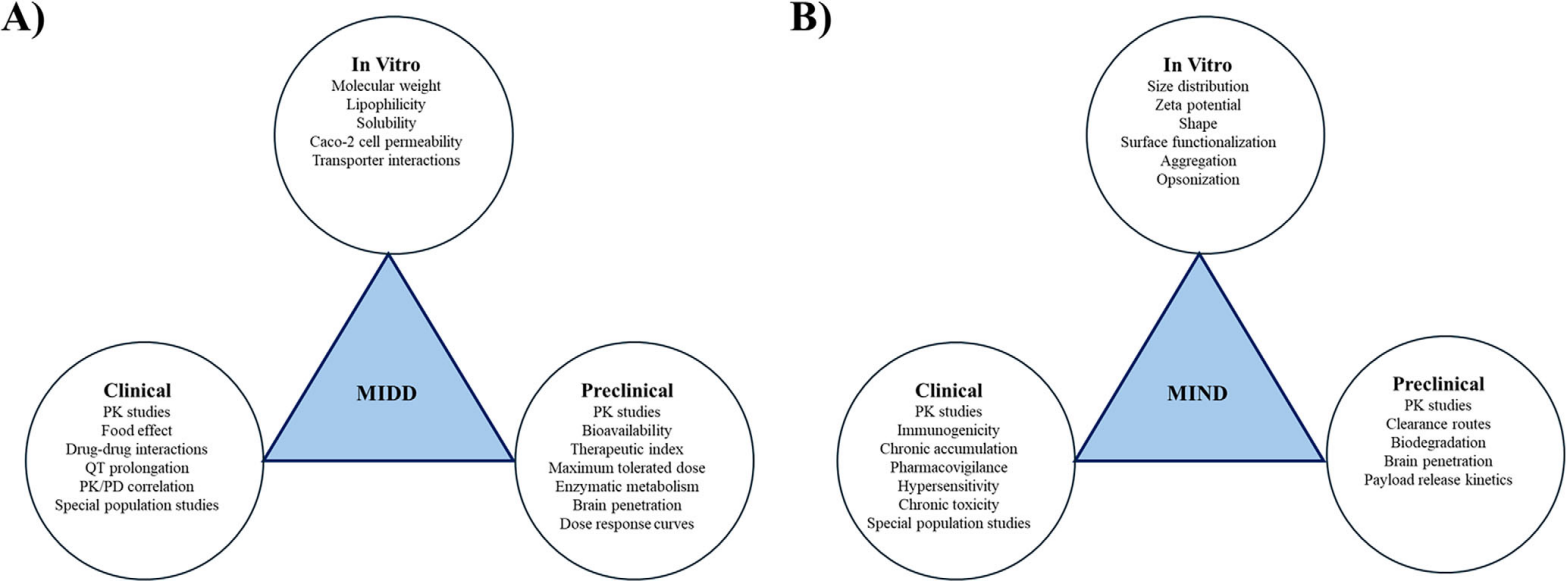
(A) Model‐informed drug discovery (MIDD) and (B) model‐informed nanoparticle discovery (MIND) workflow.

Future policy changes should continue to encourage the submission of PK models as part of investigational new drug applications (particularly for nanoparticle delivery methods), allowing for data‐driven decision‐making in dose selection, safety assessment, and clinical trial design. The basis of MIDD can be applied to nanoparticles in the form of MIND. It is the opinion of the authors that the future of nanoparticle‐based drug delivery hinges on the integration of MIND to overcome translational challenges and bridge the gap between bench and bedside. Our recommended inputs and priority areas for in vitro, preclinical, and clinical stages of both MIDD and MIND are shown in Figure 4. Addressing these challenges necessitates the adoption of advanced PK modeling techniques and mechanistic insights to optimize nanoparticle design, improve predictability, and streamline regulatory approval.

Regulatory agencies should also provide clear guidelines on the validation and acceptance of models for the development of nanoparticle‐based delivery systems, fostering greater scientific community confidence in model‐based predictions for nanoparticles. We recommend that guidelines should include simulation method, definition of statistical model performance, preclinical or clinical study reports, modeling and simulation analyses, model validation method, model analysis planning and reports, model coding scripts, as well as details of the use of drug delivery vesicle characteristics, drug characteristics, physiology, and/or disease pathophysiology. Future efforts must focus on incorporating real‐time biodistribution data, leveraging imaging technologies such as PET, and integrating omics data to build more precise and individualized models. PK modeling can bridge this gap by using preclinical intracranial injection data to build mechanistic models of nanoparticle disposition and facilitate the translation of preclinical findings to clinical applications.

In conclusion, the future of nanoparticle‐based drug delivery in part depends on a model‐informed approach that integrates advanced PK modeling, mechanistic insights, and real‐world evidence to optimize translation from bench to bedside. By addressing current gaps in PK data collection, regulation, and nanoparticle characterization, MIND can accelerate the clinical development of safer and more effective nanomedicines.

## AUTHOR CONTRIBUTIONS

**Nuo Xu:** Writing – review and editing; investigation; conceptualization; data curation; writing – original draft. **Joseph Cave:** Writing – review and editing; visualization; data curation; investigation. **Elizabeth Nance:** Conceptualization; investigation; writing – review and editing; data curation; supervision; funding acquisition; writing – original draft. **Madison Parrot:** Conceptualization; writing – original draft; visualization; writing – review and editing; investigation; data curation. **Venkata Yellepeddi:** Conceptualization; investigation; writing – review and editing; data curation; supervision; visualization; funding acquisition; writing – original draft. **Hamidreza Ghandehari:** Writing – review and editing; supervision; data curation. **Md Adnan:** Writing – review and editing; investigation; data curation; visualization.

## CONFLICT OF INTEREST STATEMENT

The authors do not have any conflicts of interest to declare.

## Supporting information


**Table S1.** Systemically administered therapeutic nanoparticle‐based drugs in clinical trials.

## Data Availability

No new data were generated or analyzed in this study. Data sharing is not applicable to this article.
