# Supplementary material for: From bench to bedside: Overcoming translational hurdles in nanoparticle research with pharmacokinetic modeling
Source: Bioeng Transl Med. 2026 Sep 8:e70176. Online ahead of print. doi: 10.1002/btm2.70176 (PMC13554393; doi:10.1002/btm2.70176)
Supplement: Supplementary file 1 [file BTM2-9999-e70176-s001.docx]

Table S1. Systemically administered therapeutic nanoparticle-based drugs in clinical trials.

| Name | Particle type/drug | Application | Clinical trial identifier |
| --- | --- | --- | --- |
| Anti-EGFR immunoliposomes | Anti-EGFR immunoliposomes loaded with doxorubicin | Solid tumors | 2007: NCT01702129 (phase 1): completed |
| NL CPT-11 | CPT-11 encapsulated liposome nanoparticle | Recurrent high-grade gliomas | 2008: NCT00734682 (phase 1): completed |
| CALAA-01 | siRNA nanoparticle | Solid tumors | 2008: NCT00689065 (phase 1): terminated |
| Nanoxel | Paclitaxel nanoparticle | Advanced breast cancer | 2009: NCT00915369 (phase 1): unknown status |
| Paclical® | Paclitaxel micellar nanoparticle | Ovarian cancer | 2009: NCT00989131 (phase 3): completed |
| Nanoparticle Albumin-Bound (Nab) | Nab paclitaxel/cyclophosphamide | Early-Stage Breast Cancer | 2010: NCT00629499 (phase 2): completed |
| Docetaxel-PNP | Polymeric nano-formulation of docetaxel | Solid tumors | 2010: NCT01103791 (phase 1): completed  2014: NCT02274610 (phase 1): completed |
|  |  |  |  |
| TKM 080301 | siRNA lipid nanoparticles | Liver cancer | 2011: NCT01437007 (phase 1): completed |
| NK105 | Paclitaxel-incorporating micellar nanoparticle | Metastatic or recurrent breast cancer | 2012: NCT01644890 (phase 3): completed |
| BIND-014 | Docetaxel nanoparticles | Urothelial carcinoma, cholangiocarcinoma, prostate/lung/cervical/head/neck cancer | 2011: NCT01300533 (phase 1): completed  2013: NCT01812746, NCT01792479 (phase 2): completed  2014: NCT02283320 (phase 2): completed  2015: NCT02479178 (phase 2): terminated |
|  |  |  |  |
|  |  |  |  |
|  |  |  |  |
|  |  |  |  |
| CRLX101 (NLG207) | 20(S)-camptothecin conjugated to cyclodextrin-polyethylene glycol-based polymer | Lung/ovarian/tubal/peritoneal/rectal/prostate cancer | 2011: NCT01380769 (phase 2): completed  2012: NCT01652079 (phase 2): completed  2013: NCT02010567 (phase 1, phase 2): terminated  2019: NCT03531827 (phase 2): terminated |
|  |  |  |  |
|  |  |  |  |
|  |  |  |  |
| EGEN-001 | IL-12 plasmid formulated with PEG-PEI-Cholesterol lipopolymer | Ovarian epithelial, fallopian tube, or primary peritoneal cancer | 2012: NCT01489371 (phase 1): completed |
| RSV-F Vaccine | RSV-F protein nanoparticle | RSV | 2012: NCT01709019 (phase 1): completed, NCT01704365 (phase 2): completed  2013: NCT01960686 (phase 2): completed  2014: NCT02296463 (phase 1): completed, NCT02247726 (phase 2): completed  2015: NCT02593071 (phase 2): completed, NCT02624947 (phase 3): completed |
|  |  |  |  |
|  |  |  |  |
|  |  |  |  |
|  |  |  |  |
|  |  |  |  |
|  |  |  |  |
| Magnetic nanoparticles | Magnetic nanoparticles | Prostate cancer | 2013: NCT02033447 (phase 1): completed |
|  |  |  |  |
| Vitamin D encapsulated in casein micelles | Vitamin D encapsulated in casein micelles | Vitamin D deficiency | 2013: NCT01807845: unknown status |
| ND-L02-s0201 | Vitamin A-coupled lipid nanoparticle containing siRNA against HSP47 | Moderate to extensive hepatic fibrosis | 2013: NCT01858935 (phase 1): completed  2014: NCT02227459 (phase 1): completed |
|  |  |  |  |
|  |  |  |  |
| TUSC2-nanoparticles | TUSC2-nanoparticles | Lung cancer | 2014: NCT01455389 (phase 1, phase 2): terminated |
| SGT-53 | Cationic liposome encapsulating DNA | Recurrent glioblastoma | 2014: NCT02340156 (phase 2): terminated |
| TargomiRs | Targeted minicells containing a microRNA mimic | Recurrent malignant pleural mesothelioma and non-small cell lung cancer | 2014: NCT02369198 (phase 1): completed |
| DCR-MYC | Double stranded RNA formulated in a liposomal nanoparticle | Solid tumors | 2014: NCT02110563 (phase 1): terminated  2015: NCT02314052 (phase 1, phase 2): terminated |
|  |  |  |  |
| NC-6004 | Cisplatin contained polymeric nanoparticle | Solid tumors | 2014:  NCT02240238 (phase 1, phase 2): completed 2016:  NCT02817113 (phase 1): terminated |
|  |  |  |  |
| BikDD nanoparticle | Cholesterol liposome-based nanoparticles | Advanced pancreatic cancer | 2015: NCT00968604 (phase 1): withdrawn |
|  |  |  |  |
| EBOV GP vaccine | Ebola virus GP nanoparticle | Ebola | 2015: NCT02370589 (phase 1): completed |
| CriPec® docetaxel | Docetaxel containing nanoparticles | Solid tumors | 2015: NCT02442531 (phase 1): completed  2018: NCT03712423 (phase 1): completed, NCT03742713 (phase 2): completed |
|  |  |  |  |
|  |  |  |  |
| NBTXR3 nanoparticle | Hafnium oxide-containing nanoparticles | Solid tumors | 2015: NCT02379845 (phase 2, phase 3): completed  2016: NCT02721056 (phase 1, phase 2): terminated  2017: NCT02805894 (phase 1, phase 2): terminated  2020: NCT04484909 (phase 1): recruiting, NCT04615013 (phase 1): recruiting  2021: NCT04505267 (phase 1): recruiting NCT04862455 (phase 2): recruiting, NCT04834349 (phase 2): withdrawn  2023: NCT05039632 (phase 1, phase 2): recruiting |
|  |  |  |  |
|  |  |  |  |
|  |  |  |  |
|  |  |  |  |
|  |  |  |  |
|  |  |  |  |
|  |  |  |  |
|  |  |  |  |
| Targeting-Enhancing Nanoparticles of Paclitaxel | Paclitaxel nanoparticles | Advanced solid tumor | 2016: NCT02979392 (phase 1): terminated |
| EP0057 | Sugar molecule cyclodextrin linked to camptothecin | Lung cancer | 2016: NCT02769962 (phase 1, phase 2): recruiting |
| PAN-301-1 (SNS-301) | Human aspartyl-asparaginyl-β-hydroxylase directed nanoparticle | Prostate cancer | 2016: NCT03120832 (phase 1): completed |
| AZD4635 | AZD4636 nanoparticle | Advanced solid malignancies | 2016: NCT02740985 (phase 1): completed |
| Phospholipovit | Nanoemulsion of phospholipids | Combined hyperlipidemia | 2016: NCT05742022 (phase 2, phase 3): completed |
| PLM60 | Mitoxantrone Hydrochloride Liposome | Non-Hodgkin's Lymphoma | 2016: NCT02856685 (phase 1, phase 2): unknown |
| SOR007 | Uncoated nanoparticle paclitaxel | Plaque psoriasis | 2016: NCT03004339 (phase 1): completed |
| MTL-CEBPA | Double stranded RNA formulated in a liposomal nanoparticle | Advanced liver cancer | 2016: NCT02716012 (phase 1): active, not recruiting |
| EGFR(V)-EDV-Dox | Doxorubicin nanoparticle | Recurrent glioblastoma | 2016: NCT02766699 (phase 1): unknown status |
| Tri-NIV | Recombinant trivalent nanoparticle | Influenza | 2017: NCT03293498 (phase 1, phase 2): completed |
| NU-0129 | Spherical nucleic acid arranged on the surface of spherical gold nanoparticles | Glioblastoma | 2017: NCT03020017 (phase 1): completed |
| NC-6300 | Nanoparticle epirubicin | Advanced solid tumors or soft tissue sarcoma | 2017: NCT03168061 (phase 1, phase 2): unknown status |
| AZD2811 | AZD2811 nanoparticle | Acute myeloid leukemia | 2017: NCT03217838 (phase 1): terminated |
| IT141 | Nanoparticle formulation of SN-38 | Advanced cancer | 2017: NCT03096340 (phase 1): terminated |
| mRNA-2416 | mRNA lipid nanoparticle | Advanced malignancies | 2017: NCT03323398 (phase 1, phase 2): terminated |
|  |  |  |  |
| STP705 | siRNA histidine-lysine co-polymer peptide nanoparticles | Hypertrophic scar | 2017: NCT02956317 (phase 1, phase 2): completed |
| MM-398 | Liposomal irinotecan | Solid tumors | 2017: NCT02631733 (phase 1): active, not recruiting  2018: NCT03337087 (phase 1, phase 2): active, not recruiting  2019: NCT03736720 (phase 1): active, not recruiting  2020: NCT03739801 (phase 1, phase 2): withdrawn  2021: NCT04753216 (phase 2): completed |
|  |  |  |  |
|  |  |  |  |
|  |  |  |  |
|  |  |  |  |
| mRNA-2752 | Lipid nanoparticle encapsulating mRNAs encoding human OX40L, IL-23, and IL-36γ | Advanced malignancies | 2018: NCT03739931 (phase 1): active, not recruiting |
| IMX-110 | Nanoparticle encapsulating a Stat3/NF-kB/poly-tyrosine kinase inhibitor | Advanced solid tumor | 2018: NCT03382340 (phase 1, phase 2): active, not recruiting |
| Quad-NIV (NanoFlu™) | Recombinant quadrivalent nanoparticle | Influenza | 2018: NCT03658629 (phase 2): terminated  2019: NCT04120194 (phase 3): completed |
|  |  |  |  |
| MTX110 | Panobinostat nanoparticle | Diffuse intrinsic pontine glioma | 2018: NCT03566199 (phase 1, phase 2): completed  2020: NCT04264143 (phase 1): completed |
|  |  |  |  |
| AGuIX® nanoparticles | Nanoparticles based on polysiloxane and gadolinium chelates | Solid tumors | 2016: NCT02820454 (phase 1): completed  2018: NCT03308604 (phase 1): recruiting  2019: NCT03818386 (phase 2): recruiting  2020: NCT04094077 (phase 2): terminated  2021: NCT04789486 (phase 1, phase 2): recruiting, NCT04899908 (phase 2): recruiting  2022: NCT04881032 (phase 1, phase 2): recruiting  2024: NCT04784221 (phase 2): not yet recruiting |
|  |  |  |  |
|  |  |  |  |
|  |  |  |  |
|  |  |  |  |
|  |  |  |  |
|  |  |  |  |
| Paclitaxel-LDE | Paclitaxel carried by a lipid nanoparticle | Atherosclerotic | 2019: NCT04148833 (phase 2, phase 3): unknown status |
| Laboratory Biomarker Analysis | Vincristine Sulfate Liposome | Recurrent Adult Acute Myeloid Leukemia | 2019: NCT02337478 (phase 2): terminated |
| MRT5201 | Codon-optimized human ornithine transcarbamylase mRNA with lipid-based nanoparticles | Ornithine transcarbamylase deficiency | 2019: NCT03767270 (phase 1, phase 2): withdrawn |
| CNM-Au8 | Gold nanocrystals | Amyotrophic Lateral Sclerosis, multiple sclerosis, Parkinson's disease | 2019: NCT04098406, NCT03815916 (phase 2): completed, NCT03993171 (phase 2): recruiting  2020: NCT03843710 (phase 2): withdrawn |
|  |  |  |  |
|  |  |  |  |
|  |  |  |  |
| Methotrexate-loaded nanoparticles | Methotrexate-loaded nanoparticles | Inflammatory reaction of Covid-19 infection | 2020: NCT04352465 (phase 1, phase 2): unknown status |
| NanoPac | Paclitaxel nanoparticle | Prostate cancer | 2020: NCT04221828 (phase 2): terminated |
| Cetuximab nanoparticle | Cetuximab loaded polymeric nanoparticles | Colon cancer | 2020: NCT03774680 (phase 1): unknown status |
| INT-1B3 | Lipid nanoparticle formulated microRNA (miR-193a-3p) mimic | Advanced solid tumor | 2020: NCT04675996 (phase 1): terminated |
| Gold factor | Gold nanoparticles | Knee joint health and function | 2020: NCT05347602: completed |
| BNT165b1 | RNA-lipid nanoparticle | Malaria | 2020: NCT05581641 (phase 1): active, not recruiting |
| AgNPs | Silver nanoparticle | Covid-19 | 2020: NCT04978025: unknown status |
| NTLA-2001 | Clustered regularly interspaced short palindromic repeats (CRISPR)/Cas9 gene editing system delivered by lipid nanoparticles | Hereditary transthyretin amyloidosis with polyneuropathy, cardiomyopathy, or wild type cardiomyopathy | 2020: NCT04601051 (phase 1): active, not recruiting |
| ARCT-810 | mRNA coding for cystic fibrosis transmembrane conductance regulator (CFTR) protein formulated in a lipid nanoparticle | Ornithine transcarbamylase deficiency | 2020: NCT04416126 (phase 1): completed, NCT04442347 (phase 1): active, not recruiting  2022: NCT05526066 (phase 2): recruiting |
|  |  |  |  |
|  |  |  |  |
| MTX-LDE | Methotrexate carried by a lipid nanoparticle | Covid-19, atherosclerotic | 2020:  NCT04610567 (phase 1, phase 2): unknown status  NCT04616872 (phase 2, phase 3): unknown status |
|  |  |  |  |
| GBP510 | SK SARS-CoV-2 recombinant protein nanoparticle | Covid-19 | 2020: NCT04368988 (phase 1, phase 2): completed  2021: NCT04742738 (phase 1, phase 2): completed, NCT04750343 (phase 1, phase 2): active, not recruiting, NCT05007951 (phase 3): active, not recruiting  2022: NCT05175950 (phase 2): active, not recruiting |
|  |  |  |  |
|  |  |  |  |
|  |  |  |  |
|  |  |  |  |
| EP0057 | Nanoparticle-drug conjugate | Advanced gastric and ovarian cancer, small cell lung cancer | 2020: NCT04669002 (phase 2): completed  2023: NCT05411679 (phase 2): withdrawn |
|  |  |  |  |
| PRECIOUS-01 | Invariant natural killer T cell (iNKT) activator threitolceramide-6 (ThrCer6, IMM60) and the New York esophageal squamous cell carcinoma-1 (NY-ESO-1) cancer-testis antigen peptides encapsulated in PLGA nanoparticles | Advanced solid tumor | 2021: NCT04751786 (phase 1): recruiting |
| ChulaCov19 vaccine | SARS-CoV-2 wild type S-spike mRNA lipid nanoparticle | Covid-19 | 2021: NCT04566276 (phase 1, phase 2): completed |
| CoV2 SAM | Self-amplifying mRNA lipid nanoparticle | Covid-19 | 2021: NCT04758962 (phase 1): completed |
| SpFN | SARS-CoV-2-spike-ferritin nanoparticle | Covid-19 | 2021: NCT04784767 (phase 1): unknown status |
| DS-5670a | mRNA lipid nanoparticles | Covid-19 | 2021: NCT04821674 (phase 1, phase 2): completed |
| UFluA | Influenza A hemagglutinin stabilized stem nanoparticle | Influenza | 2021: NCT05155319 (phase 1): completed |
| ICC vaccine | Influenza quadrivalent hemagglutinin nanoparticle and SARS-CoV-2 rS nanoparticle | Covid-19, influenza | 2021: NCT04961541 (phase 1, phase 2): completed  2022: NCT05519839 (phase 2): active, not recruiting |
|  |  |  |  |
| PepGNP | Gold nanoparticle plus peptides | Dengue virus, Covid-19 | 2021: NCT04935801 (phase 1): completed  2022: NCT05113862 (phase 1): completed  2023: NCT05633446 (phase 1, phase 2): not yet recruiting |
|  |  |  |  |
|  |  |  |  |
| Quaratusugene ozeplasmid (Reqorsa) | DNA plasmid with the TUSC2 tumor suppressor gene encapsulated lipid nanoparticles | Non-small cell lung cancer | 2021: NCT04486833 (phase 1, phase 2): recruiting  2022: NCT05062980 (phase 1, phase 2): recruiting  2024: NCT05703971 (phase 1, phase 2): recruiting |
|  |  |  |  |
|  |  |  |  |
| Nano-QUT | Quercetin-encapsulated PLGA-PEG nanoparticles | Oral cancer | 2022: NCT05456022 (phase 2): not yet recruiting |
| Carbon nanoparticle-loaded iron [CNSI-Fe(II)] | Carbon nanoparticle-loaded iron | Advanced solid tumor | 2022: NCT06048367 (phase 1): recruiting |
| WGI-0301 | Lipid Nanoparticle Suspension of Akt-1 Antisense Oligonucleotide | Advanced solid tumors or non-small cell lung, biliary and bladder cancer | 2022: NCT05267899 (phase 1): recruiting |
| BL 1.33 | Bupivacaine Liposome | Osteoarthritis of the shoulder | 2022: NCT04134442 (phase 4): withdrawn |
| GF-AuKHC46 | Gold Factor (gold nanoparticles, AuNPs) | Knee Osteoarthritis | 2022: NCT05347602 (phase 1, phase 2, phase 3): completed |
| mRNA-3745 | Lipid nanoparticle | Glycogen storage disease | 2022: NCT05095727 (phase 1): recruiting |
| OTX-2002 | mRNA lipid nanoparticles | Solid tumor | 2022: NCT05497453 (phase 1, phase 2): recruiting |
| HDT-301 | Replicon RNA (repRNA) SARS-CoV-2 nanoparticle | Covid-19 | 2022: NCT04844268 (phase 1): recruiting, NCT05132907 (phase 1): active, not recruiting |
| QTP104 | repRNA lipid-inorganic nanoparticle | Covid-19 | 2022: NCT05876364 (phase 1): active, not recruiting |
| Covovax® | Recombinant spike protein nanoparticle | Covid-19 | 2022: NCT05112848 (phase 2): completed, NCT05433285 (phase 3): completed |
|  |  |  |  |
| Epstein-Barr Virus (EBV) gp350-Ferritin Vaccine | Helicobacter pylori non-heme ferritin fused to EBV gp350 which self-assembles to nanoparticles | EBV infection | 2022: NCT04645147 (phase 1): active, not recruiting  2023: NCT05683834 (phase 1, phase 2): recruiting |
|  |  |  |  |
| RSV mRNA vaccine | RSV mRNA lipid nanoparticles | RSV | 2022: NCT05639894 (phase 1, phase 2): active, not recruiting  2024: NCT06237296 (phase 1): not yet recruiting |
|  |  |  |  |
|  |  |  |  |
| V3G CH848 Pr-NP1 | mRNA lipid nanoparticles | HIV | 2023: NCT05903339 (phase 1): recruiting |
| OP-101-004 | OP-101 (Dendrimer N-acetyl-cysteine) | COVID-19 | 2023: NCT04458298 (phase 1): terminated |
| D-4517.2 | Hydroxyl dendrimer VEGFR tyrosine kinase inhibitor | Neovascular age-related macular degeneration / Diabetic macular edema | 2023: NCT05387837 (phase 2): recruiting |
| DCVC H1 HA mRNA vaccine | DCVC H1 HA mRNA lipid nanoparticle | Influenza | 2023: NCT05945485 (phase 1): recruiting |
| HA mRNA vaccines | mRNA lipid nanoparticles | Influenza | 2023: NCT05829356 (phase 1): active, not recruiting |
| H1ssF-3928 | mRNA lipid nanoparticles | Influenza | 2023: NCT05755620 (phase 1): recruiting |
| BNT165e | RNA-lipid nanoparticles | Malaria | 2023: NCT06069544 (phase 1, phase 2): recruiting |
| PLZ4-coated paclitacel-loaded micelles (PPM) | PPM | Recurrent or refractory non-muscle invasive bladder cancer | 2023: NCT06173349 (phase 1): recruiting |
| Irinotecan liposome injection | Convection enhanced delivery (CED) of nanoliposomal irinotecan (nal-IRI) | Diffuse intrinsic pontine glioma | 2023: NCT03086616 (phase 1): completed |
| Nanoparticle Albumin-Bound (Nab) | Nab-paclitaxel/Rituximab-coated Nanoparticle AR160 | Relapsed or Refractory B-Cell Non-Hodgkin Lymphoma | 2023: NCT03003546 (phase 1): completed |
| ARCT-032 | mRNA coding for OTC formulated in a lipid nanoparticle | Cystic fibrosis | 2023: NCT05712538 (phase 1): recruiting |
|  |  |  |  |
| TI-0010 | circRNA lipid nanoparticle | Covid-19 | 2023: NCT06205524 (phase 1): recruiting |
| BIO 300 oral suspension | Genistein nanoparticles | Covid-19 | 2023: NCT04482595 (phase 2): recruiting |
| XBB.1.5 Vaccine | SARS-CoV-2 rS protein nanoparticle | Covid-19 | 2023: NCT05925127 (phase 2, phase 3): active, not recruiting, NCT05975060 (phase 2, phase 3): recruiting |
|  |  |  |  |
| PTX-COVID19-B | mRNA lipid nanoparticles | Covid-19 | 2023: NCT05534035, NCT05534048 (phase 3): not yet recruiting |
|  |  |  |  |
| MT-302 | TROP2-FcA mRNA lipid nanoparticle | Advanced or metastatic epithelial tumors | 2023: NCT05969041 (phase 1): recruiting |
| RNA-nanoparticle vaccine | Autologous total tumor mRNA loaded DOTAP liposome | Melanoma | 2024: NCT05264974 (phase 1): recruiting |

siRNA: small interfering RNA. Nab: Nanoparticle Albumin-Bound. RSV: Respiratory Syncytial Virus. GP: glycoprotein. mRNA: messenger RNA. HIV: Human Immunodeficiency Virus. circRNA: Circular RNA.
